# Gene networks governing the response of a calcareous sponge to future ocean conditions reveal lineage‐specific *XBP1* regulation of the unfolded protein response

**DOI:** 10.1002/ece3.11652

**Published:** 2024-06-30

**Authors:** Niño Posadas, Cecilia Conaco

**Affiliations:** ^1^ Marine Science Institute, University of the Philippines Diliman Quezon City Philippines; ^2^ Present address: Centre for Chromosome Biology, School of Biological and Chemical Sciences University of Galway Galway Ireland

**Keywords:** Calcarea, climate change, gene duplication, transcription factor, UPR

## Abstract

Marine sponges are predicted to be winners in the future ocean due to their exemplary adaptive capacity. However, while many sponge groups exhibit tolerance to a wide range of environmental insults, calcifying sponges may be more susceptible to thermo‐acidic stress. To describe the gene regulatory networks that govern the stress response of the calcareous sponge, *Leucetta chagosensis* (class Calcarea, order Clathrinida), individuals were subjected to warming and acidification conditions based on the climate models for 2100. Transcriptome analysis and gene co‐expression network reconstruction revealed that the unfolded protein response (UPR) was activated under thermo‐acidic stress. Among the upregulated genes were two lineage‐specific homologs of X‐box binding protein 1 (*XBP1*), a transcription factor that activates the UPR. Alternative dimerization between these *XBP1* gene products suggests a clathrinid‐specific mechanism to reversibly sequester the transcription factor into an inactive form, enabling the rapid regulation of pathways linked to the UPR in clathrinid calcareous sponges. Our findings support the idea that transcription factor duplication events may refine evolutionarily conserved molecular pathways and contribute to ecological success.

## Graphical abstract

This study describes the gene regulatory networks that govern the stress response of a calcareous sponge under future ocean scenarios. We discovered that the sponge activates both adaptive and pro‐apoptotic phases of the unfolded protein response (UPR) under combined temperature and pH stress. Keeping the balance between these two UPR phases in clathrinid calcisponges may be mediated by the expression of two X‐box binding protein 1 (*XBP1*) homologs that are predicted to produce dimer pairs with varying DNA‐binding properties.

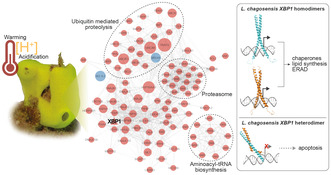

## 1 INTRODUCTION

Calcifying members of the reef biome have transcended major upheavals throughout Earth's history and played crucial roles in shaping marine ecosystems (Hull, 2017). However, rising anthropogenic CO_2_ emissions are predicted to acidify oceans at an accelerated rate (MacFarling Meure et al., 2006). Marine calcifiers or organisms that build calcite or aragonite skeletons, are particularly vulnerable to changes in seawater carbonate chemistry (Alter et al., 2024). Historical data shows that increasing ocean *p*CO_2_ over the past 40,000 years has led to a decline in calcification of the dominant coccolithophore, *Emiliania huxleyi* (Beaufort et al., 2011). Similar impacts have been reported for other heavily calcified organisms, such as calcifying algae, corals, mollusks, and echinoderms (Kroeker et al., 2013). Although some studies suggest that the impact of ocean acidification may not be as severe (Leung et al., 2022), the co‐occurrence of acidification with warming could exacerbate detrimental effects (Alter et al., 2024; Pörtner et al., 2005; Prada et al., 2017). This is evidenced by reduced thermal tolerance of the sea urchin, *Loxechinus albus*, under acidified conditions (Manriquez et al., 2019), and the decline in calcification rates of scleractinian corals, *Pocillopora damicornis* and *Stylophora pistillata*, subjected to elevated temperature and lowered pH (31°C, pH 7.55) (Guillermic et al., 2021). On the other hand, some studies suggest that physiological buffering by microalgal symbionts may ameliorate the interactive effects of warming and acidification in certain species of corals and foraminiferans (Doo et al., 2020; Prada et al., 2017). These contrasting outcomes underscore the complexity of responses of different organisms to future ocean conditions, with impacts determined by varied physiological traits among taxonomic groups (Kroeker et al., 2013). Despite these conflicting reports, the responses of other marine calcifiers, particularly calcareous sponges, remain poorly understood.

Sponges (Phylum Porifera) are sessile aquatic invertebrates characterized by a filter‐feeding lifestyle. They are among the oldest multicellular animals, having diverged from other metazoans during the Precambrian around 600 MYA (Yin et al., 2015). Due to their early‐branching position, sponges have been instrumental in elucidating the origins and early evolution of animal complexity (Philippe et al., 2011; Pick et al., 2010; Taylor et al., 2007). Marine sponges are critical members of the benthos, playing major roles in ecosystem structuring, reef consolidation, bio‐erosion, and nutrient cycling (Bell, 2008). A comprehensive review of climate change‐associated studies proposed that sponges are to be winners under future climate scenarios (Bell et al., 2018). Their remarkable ecological success across habitats and environments is attributed to their simple yet highly adaptable body plan (Carballo & Bell, 2017; Van Soest et al., 2012). However, recent studies have also demonstrated the vulnerability of select sponge species to climate change‐associated stressors (Chai et al., 2024; Perkins et al., 2022; Rondon et al., 2020; Stevenson et al., 2020).

There are four morphologically distinct extant sponge classes (i.e., Demospongiae, Hexactinellida, Homoscleromorpha, and Calcarea) (Van Soest et al., 2012). Calcareans, which are distinguished from the other sponge classes by their calcified structures (Sethmann & Wörheide, 2008), are grouped into two monophyletic subclasses: the Calcinea (composed of order Clathrinida) and the Calcaronea (composed of orders Baeriida, Leucosolenida, and Lithonida) (Manuel et al., 2003). Calcareous sponges are predicted to be more vulnerable to thermo‐acidic stress due to their calcium carbonate skeletons, lower microbiome diversity, and rapid suppression of host immune functions under stress (Posadas et al., 2022). However, recent findings show that some calcaronean calcisponges, such as *Leucosolenia complicata*, *Paraleucilla magna*, and *Grantia* sp., can tolerate low pH conditions (McCullough et al., 2024; Peck et al., 2015; Ribeiro et al., 2023). To date, only two studies have examined the combined effects of warming and acidification stress on the microbiome and spicules of calcareous sponges (Posadas et al., 2022; Ribeiro et al., 2020). This highlights the need for further research to investigate the compounded impacts of thermo‐acidic stress on calcareans and to identify other processes underpinning their response to stress signals.

Next‐generation sequencing and systems biology are powerful approaches enabling elucidation of molecular mechanisms engaged in the cellular stress response of non‐model systems (Fan et al., 2024; Jia et al., 2021). For example, transcriptome sequencing of the massive starlet coral, *Siderastrea siderea*, revealed activation of biocalcification‐related processes under high *p*CO2 and repression of functions related to environmental sensing under thermal stress (Davies et al., 2016). Similarly, transcriptome sequencing of the rice coral, *Montipora capitata*, identified transcription factors that were key in regulating its thermal stress response (Shumaker et al., 2019). In addition, differential gene expression analysis of demosponges, *Haliclona tubifera* and *Spongia officinalis*, subjected to elevated temperatures revealed activation of molecular chaperones and other proteins involved in mitigating endoplasmic reticulum (ER) stress and operating the unfolded protein response (UPR) (Guzman & Conaco, 2016; Koutsouveli et al., 2020).

The UPR is an evolutionarily conserved signal transduction pathway that is critical for the maintenance and restoration of ER homeostasis, especially under stress conditions (Karagoz et al., 2019). This well‐orchestrated response network detects the build‐up of aberrant proteins in the ER and enforces adaptive mechanisms by optimizing protein synthesis and folding, as well as initiating ER‐associated protein degradation pathways (Walter & Ron, 2011). When ER imbalance remains unmitigated due to persistent protein aggregation and sustained stress conditions, the UPR switches from the adaptive or pro‐survival phase to the pro‐apoptotic phase, which promotes cell death in affected tissues (Chen & Brandizzi, 2013; Walter & Ron, 2011). The balance between the adaptive and pro‐apoptotic phases of UPR is regulated by X‐box binding protein 1 (*XBP1*), a UPR‐specific transcription factor, in coordination with C/EBP homologous protein (*CHOP*) and c‐Jun NH2‐terminal kinase (*JNK*) (Chan et al., 2015).

*XBP1* belongs to the basic region leucine zipper (bZIP) family, one of the largest and most diverse classes of transcription factors among eukaryotes (de Mendoza et al., 2013). Phylogenetic analysis indicates that the bZIP protein superfamily arose from a single ancestral eukaryotic gene and underwent multiple independent expansions in multicellular lineages (Jindrich & Degnan, 2016). bZIP transcription factors form dimers that bind to dsDNA (Bader & Vogt, 2006). Dimerization significantly increases both the affinity and recognition specificity of transcription factors to avoid spurious DNA binding and transcription (Georges et al., 2010; Todeschini et al., 2014). In effect, the choice of protein partner diversifies bZIP targets and regulatory functions (Amoutzias et al., 2008). Conserved and novel bZIPs have been reported to play key roles in the stress response and improved tolerance in plants (e.g., rice (Yang et al., 2019), *Arabidopsis* (Noh et al., 2021), and wheat (Agarwal et al., 2019)) and animals (e.g., coral (Dimos et al., 2019), fruit fly (Brown et al., 2021), and nematode (Lazetic et al., 2022)).

In this study, we sequenced the transcriptome of *Leucetta chagosensis* (class Calcarea, order Clathrinida, family Leucettidae) subjected to elevated temperature and reduced pH treatments to describe the molecular mechanisms and key regulators underlying the stress response of a calcareous sponge under future ocean conditions. This revealed the activation of the UPR network under thermo‐acidic stress and the potential role of two lineage‐specific homologs of *XBP1* in the rapid regulation of UPR in clathrinid calcareous sponges. These findings highlight how gene duplication events may fine‐tune evolutionarily conserved molecular pathways and contribute to ecological success.

## 2 MATERIALS AND METHODS

### 2.1 Sponge collection and culture

The work described here is part of a larger study investigating the response of the *L. chagosensis* holobiont to thermo‐acidic stress (Posadas et al., 2022). Six specimens of *L. chagosensis* were collected from the Bolinao‐Anda Reef Complex in Pangasinan, northwestern Philippines (16.296°N, 120.014°E) with permission from the Philippines Department of Agriculture (Gratuitous Permit No. 0169‐19). Sponge identities were confirmed by their morphology (Hooper & Van Soest, 2002) and 28S ribosomal RNA gene analyses (Posadas et al., 2022). Donor sponges were cut into 12 (≈1 cm^3^) fragments using a sterile razor and allowed to heal in situ for 30 days. Healed fragments were brought to the Bolinao Marine Laboratory and reared for 7 days in aquaria receiving flow‐through seawater under ambient conditions (pH 8.0 and 28°C).

### 2.2 Stress response experiments

Stress response experiments were conducted as previously described (Posadas et al., 2022). Conditions in experimental aquaria were manipulated to levels designed to simulate the present day and predicted 2100 Representative Concentration Pathway (RCP) 6.0 and 8.5 scenarios (Pachauri et al., 2014). Treatment conditions include: (i) pH 8.0, 28°C (Present Day), (ii) pH 7.6, 28°C (Acidification), (iii) pH 8.0, 32°C (Warming), (iv) pH 7.8, 30°C (RCP 6.0), and (v) pH 7.6, 32°C (RCP 8.5). Temperatures were regulated using 300 W submersible heaters (EHEIM GmbH & Co. KG, Baden Wurttemberg, Germany), levels of injected CO_2_ were manipulated using a mass flow controller, and illumination was provided by daylight LED lamps following a 12:12 light:dark photoperiod. Light and temperature in the tanks were monitored using submersible loggers (HOBO pendant, Onset Computer Corp., Bourne, MA, USA), pH was measured using a SevenGo Duo Pro pH meter (Mettler Toledo, Columbus, OH, USA), and dissolved oxygen and salinity were measured using a multiparameter meter (Pro 2030; YSI Inc., Yellow Springs, OH, USA).

Each treatment was represented by four independent replicate aquaria containing three fragments, with each fragment originating from a different sponge donor (*n* = 12 fragments per treatment). Temperature and pH levels were changed gradually (temperature: +1°C/day, pH: −0.5/day) until the desired conditions were reached. Treatment conditions were maintained for 2 days (48 h) then the experiment was terminated because tissue necrosis had begun to manifest in some fragments, with visible whitening and disintegration of tissues. Surviving sponges were washed with ultraviolet‐filtered seawater, any necrotic tissues were excised, and the remaining healthy tissues were flash‐frozen in liquid nitrogen for transport, then stored at −80°C.

### 2.3 Transcriptome sequencing, assembly, and annotation

Total RNA of sponge individuals that were collected after 2 days (48 h) of sustained exposure to the treatments was extracted from 50 to 100 mg of tissues using TRIzol (Invitrogen, Waltham, MA, USA) following the manufacturer's protocol. Libraries were prepared from three samples per treatment, except for the Warming and RCP 6.0 treatments, for which only two samples each had high‐quality RNA extracts. Replicate samples for each treatment were selected from different donor sponges. Barcoded libraries were prepared at Macrogen, South Korea, using the Truseq RNA Library Preparation Kit (Illumina, Inc.). mRNA‐enriched libraries were sequenced on the Novaseq 6000 platform (Illumina, Inc.) to generate 100 bp paired‐end reads. Transcriptome assembly and annotation for *L. chagosensis* was initially reported in (Posadas et al., 2022) (details in Supplementary Methods, Tables S1–S3). The assembly is comparable to other sponge species in terms of quality and gene content (Supplementary Methods and Results, Figures S1, S2, Tables S4–S6). The reference transcriptome used in this study is available at DDBJ/EMBL/GenBank under the accession number GIYV00000000. Raw sequence reads can be accessed from the NCBI Short Read Archive database under BioProject PRJNA689294.

### 2.4 Gene co‐expression network analysis

Gene co‐expression networks were reconstructed from rlog‐transformed expected counts through weighted gene co‐expression network analysis or WGCNA (Langfelder & Horvath, 2008) implemented in R. WGCNA identifies sets of genes, called modules, which have similar expression patterns across treatments. Lowly expressed genes (<10 counts in at least two libraries) were filtered out prior to the analysis. Only 22,417 *L. chagosensis* genes were used for network construction, with the following parameters: softPower = 30 and minModuleSize = 30. To robustly identify both positive and negatively correlated genes, we implemented csuWGCNA (Dai et al., 2019), which combines signed and unsigned methods of WGCNA, for sensitive module detection. Modules with similar expression patterns were further clustered (cutHeight = 0.60) to generate the final set of modules used in downstream analyses.

The general expression pattern of genes in a module is represented by its eigengene. Correlation of module eigengenes with treatment conditions identified modules representing genes involved in the response of *L. chagosensis* to differing intensities of warming and acidification stress. Genes within a module were characterized by gene significance (GS), representing the correlation between an individual gene and the treatment of interest, and module membership (MM), representing the correlation of the individual gene's expression with the module eigengene (Langfelder & Horvath, 2008). Hub genes, which likely serve as regulatory components of transcriptional networks (Fabina et al., 2013), were identified as genes that were significantly associated with a specific treatment (GS ≥ |0.2|) and that were highly co‐expressed (MM > |0.8|) within a module.

Differentially expressed genes were identified using the edgeR (Robinson et al., 2010) package in R. Genes were considered differentially expressed if upregulation or downregulation was ≥4‐fold relative to the controls with a False Discovery Rate (FDR) ≤ 0.05. Pairwise comparisons were conducted between the Present Day samples and samples subjected to the other treatments (Supplementary Methods).

### 2.5 Pfam, gene ontology, and pathway enrichment analysis

Pfam and GO enrichment analyses for module members and differentially expressed genes were performed using a script (github.com/fle1/canolab_scripts/blob/master/Pfam_enrichment.R) and the topGO package (Alexa & Rahnenführer, 2009) implemented in R. Only Pfam domains and GO terms with *p*‐value < 0.05 were considered significantly enriched.

Hub genes were annotated against the human proteome v.11.5 from the STRING v.11 database (von Mering et al, 2003) using Blastp with an *e*‐value cut‐off of 1 × 10^−5^. Top hits for each hub gene were used as input in KEGG pathway enrichment analysis. Enriched pathways were summarized into general categories based on KEGG pathway maps. Protein–protein interactions of genes involved in enriched pathways (score > 0.40 and FDR < 0.01) were retrieved from the STRING v.11 database (von Mering et al., 2003). Interaction networks were visualized using Cytoscape v.3.7.2 (Shannon et al., 2003). Relative expression of sponge gene homologs in RCP 8.5 relative to the Present Day control was computed as the average sum of transcripts per million (TPM).

### 2.6 Phylogenetic analysis of *XBP1* homologs

Homologs of *XBP1* genes in the UniProtKB/Swiss‐Prot database were identified in a broad selection of organisms using Blastp (*e*‐value ≤ 1 × 10^−5^) (Table S7). Sequences corresponding to the bZIP_1 domain (PF00170) of *XBP1* were aligned using Clustal Omega (Madeira et al., 2019) and the alignment was manually trimmed (Data S1). The best‐fit substitution model (LG + I + G) was identified based on Bayesian Information Criterion using prottest v3.4.2 (Darriba et al., 2011). Bayesian inference analysis was performed in MrBayes v.3.2 (Ronquist & Huelsenbeck, 2003) with two‐independent MCMC runs and four chains per run. The analysis was sampled every 100 trees until the average standard deviation of split frequencies was < 0.01. The first 25% of trees were discarded as burn‐in.

### 2.7 Expression of *XBP1* duplicates

To confirm the expression of *XBP1* genes in *L. chagosensis*, full‐length and bZIP domains of *LchaXBP1_1* and *LchaXBP1_2* were amplified from cDNA libraries. Expression levels of *LchaXBP1* homologs in Present Day and RCP 8.5 samples were determined by quantitative PCR (Supplementary Methods, Table S8).

### 2.8 Prediction of *XBP1* dimer structures and DNA binding potential

Leucine zipper heptads of *LchaXBP1*, *PoriXBP1*, and *CspXBP1* homologs were manually evaluated to identify possible dimerization pairs based on the presence of specific amino acids at positions that regulate dimerization stability and specificity (Figure S3). Predicted 3D models of *XBP1* dimer pairs were built through SWISS‐MODEL (Waterhouse et al., 2018). Best‐hit structural homologs based on the QMEANDisCO score were used as templates for predicting the protein structure. QMEANDisCO is a composite scoring function derived from the entire structure and per residue quality estimates (0, lowest – 1, highest).

*XBP1* is known to preferentially bind the CRE‐like element in which the core “ACGT” is highly conserved (Clauss et al., 1996). To test the binding potential of predicted *L. chagosensis XBP1* dimer pairs, we performed in silico docking experiments using the CRE DNA substrate containing “aureo‐box” TGACGT (Banerjee & Mitra, 2020) from CREB structure PDB ID: 1DH3 (Schumacher et al., 2000) as ligand. The best‐scoring complex structures based on pyDock (Rodriguez‐Lumbreras et al., 2022) and FTDOCK (Gabb et al., 1997) algorithms were visualized and post‐processed in Pymol (DeLano, 2002) and PDBePISA (Krissinel & Henrick, 2007) to identify *XBP1* – CRE interfaces.

### 2.9 Visualization

All visualizations were done using ggplot2 (Wickham, 2016) in R. Phylogenetic trees were visualized in iTOL (Letunic & Bork, 2019).

## 3 RESULTS

To describe the responses of a calcareous sponge to future ocean conditions, *L. chagosensis* individuals were subjected to stress response experiments with warming and acidification conditions based on the climate models for 2100: (i) pH 8.0, 28°C (Present Day), (ii) pH 7.6, 28°C (Acidification), (iii) pH 8.0, 32°C (Warming), (iv) pH 7.8, 30°C (RCP 6.0), and (v) pH 7.6, 32°C (RCP 8.5). As described in our previous study, *L. chagosensis* exhibited visible tissue necrosis (i.e., whitening and disintegration of tissues) under the Warming, RCP 6.0, and RCP 8.5 conditions, but not in the Acidification only treatment, and only 25% (3 out of 12) of the *L. chagosensis* fragments survived in the RCP 8.5 treatment after 2 days (48 h) of sustained exposure (Posadas et al., 2022).

### 3.1 Transcriptome of *L. chagosensis* is dynamic under stress

**FIGURE 1:**
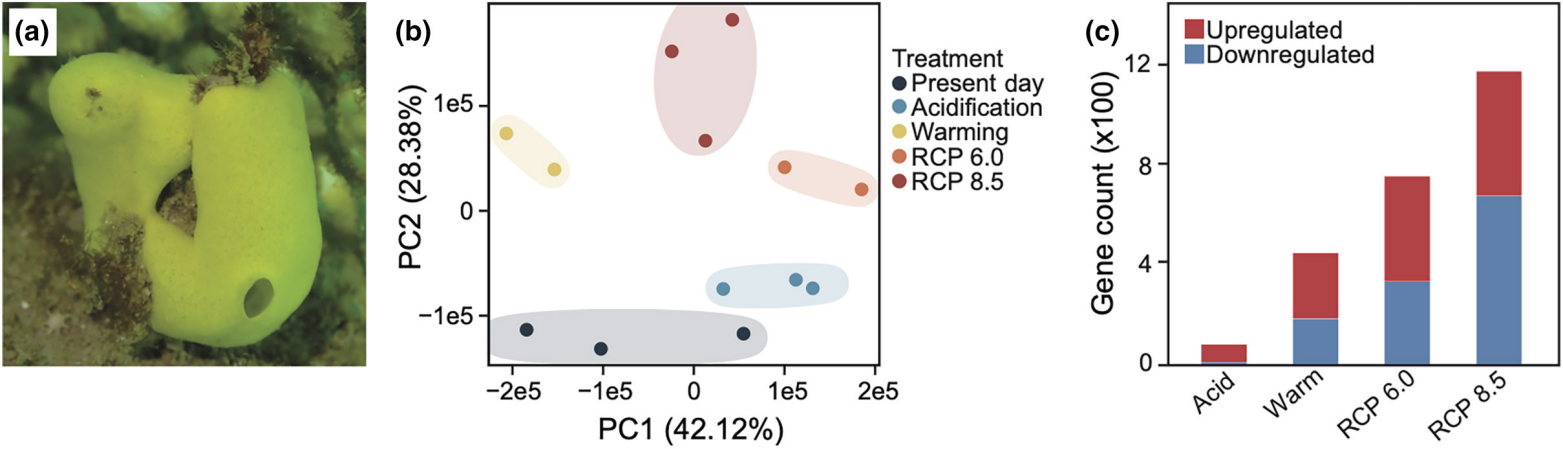
Transcriptome dynamics in *Leucetta chagosensis* under stress. (a) *Leucetta chagosensis* in its natural habitat. (b) Transcriptome‐wide changes in the sponge subjected to various treatments visualized through principal component analysis. Ellipses were computed using the Khachiyan algorithm (tolerance level = 0.01). (c) Number of differentially regulated genes (log_2_ fold change ≥2, FDR ≤0.05) in each treatment relative to Present Day controls.

We sequenced the transcriptome of *L. chagosensis* (Figure 1) to describe gene expression dynamics accompanying the response to thermo‐acidic stress (Supplementary Results).

Principal component analysis (PCA) and pairwise comparisons of transcriptome profiles of control (Present Day) and treated samples revealed that the host transcriptome of *L. chagosensis* is dynamic under stress (Figure 1b). Two PC axes explained 70.50% of variance, with the first axis (42.12%) and second axis (28.38%) correlating to the effects of pH and temperature, respectively. The number of differentially expressed genes across treatments varied, with the highest in RCP 8.5 (*n* = 1194), followed by RCP 6.0 (*n* = 766), Warming (*n* = 454), and Acidification (*n* = 80) (Figure 1c). A notable number of upregulated genes across the four treatments have detectable paralogs (Acidification = 48, Warming = 163, RCP 6.0 = 244, RCP 8.5 = 270) (Figure S4) and are species‐specific (Acidification = 12, Warming = 80, RCP 6.0 = 122, RCP 8.5 = 170) (Figure S5).

### 3.2 Gene regulatory networks governing the sponge stress response

**FIGURE 2:**
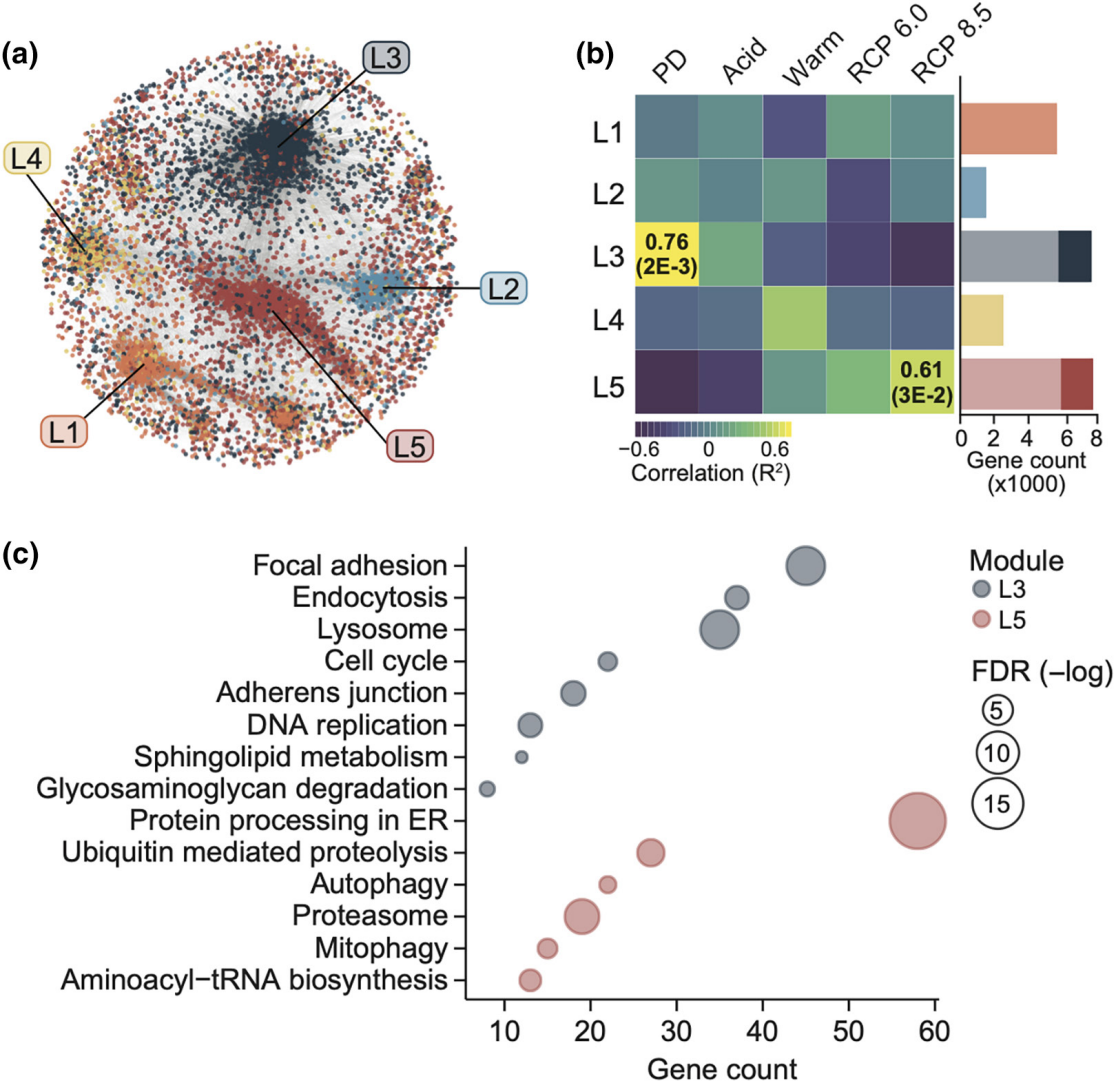
Gene networks governing the response of *Leucetta chagosensis* to thermo‐acidic stress. (a) Co‐expression network of highly expressed genes in *L. chagosensis*. Each node represents a single gene. Edge distance indicates co‐expression strength between genes. (b) Correlation of gene modules (L1–L5) with the five treatments. Values are indicated for significant positive correlations. Bar graph (right) shows the number of genes per module, with hub genes represented by the darker shades. Acid, acidification; PD, present day; Warm, warming. (c) KEGG pathway enrichment analysis for module L3 and L5 hub genes.

To describe the gene regulatory networks (GRNs) that govern the calcareous sponge stress response, highly co‐expressed genes (i.e., gene modules) in *L. chagosensis* were identified using WGCNA (Figure 2a). Five gene co‐expression modules were identified, two of which showed significant correlation with the tested conditions (Figure 2b). Module L3 was associated with Present Day (*R* ^2^ = 0.76, *p*‐value = .002), and contained 7,582 genes that were generally downregulated with stress (Figure 2b, Figure S6). Module L5 was associated with RCP 8.5 (*R* ^2^ = 0.61, *p*‐value = .03) and contained 5,803 genes that were activated with stress (Figure 2b, Figure S6). The other modules did not show significant correlations with treatment conditions. Module L3 included 2577 hub genes with 1584 (61.46%) that were annotated with the STRING v.11 database, while module L5 had 1849 hub genes with 1346 (72.08%) annotated (Figure 2b, Table S9). We focused on these annotated hub genes in subsequent analyses.

### 3.3 Pathways activated or repressed under stress

KEGG pathway enrichment analysis revealed that L3 hub genes, which were downregulated with stress, are enriched for players involved in cell growth and death (hsa4110: cell cycle), replication and repair (hsa3030: DNA replication), transport and catabolism (hsa4142: lysosome, has4144: endocytosis), glycan metabolism (hsa531: glycosaminoglycan degradation), lipid metabolism (hsa600:sphingolipid metabolism), and cellular community (hsa4510: focal adhesion, hsa4520: adherens junction) (Figure 2c). Repression of these basic cellular and metabolic processes, as well as ECM components, suggest that *L. chagosensis* is likely susceptible to prolonged exposure to thermo‐acidic stress, as predicted for other calcareous sponges (Bell et al., 2018; Guzman & Conaco, 2016; Smith et al., 2013). In fact, genes implicated in transcriptional control, cell proliferation and extracellular matrix maintenance, biocalcification, metabolism, stress response, and innate immunity were differentially regulated under RCP 8.5 (Table S10). Downregulated genes included epithelial and focal adhesion components (collagen (*CO4A1*, *CO1A2*, *CO6A5*, *CTHR1*, *COLL2*, and *COLL5*), integrin (*ITB6*), fibrillin (*FBN2* and *FBN3*), and mucin (*MUC5B* and *MLP*)), along with biocalcification genes, such as carbonic anhydrases (*CAH1*, *CAH2*, and *CAH7*) and bicarbonate transporter proteins (Tables S11, S12), which may have compromised the integrity of *L. chagosensis* physical defenses and structural support under thermo‐acidic conditions.

Hub genes in module L5, which were upregulated with stress, are enriched for pathway components engaged in transport and catabolism (hsa4137: mitophagy and hsa4140: autophagy), translation (hsa970: aminoacyl‐tRNA biosynthesis), and protein folding, sorting, and degradation (hsa4141: protein processing in endoplasmic reticulum, hsa3050: proteasome, hsa4120: ubiquitin‐mediated proteolysis) (Figure 2c). These pathways are part of or linked to the UPR, a well‐orchestrated signal transduction network that serves to restore normal ER functions under stress (Karagoz et al., 2019).

### 3.4 Activation of the unfolded protein response under stress

**FIGURE 3:**
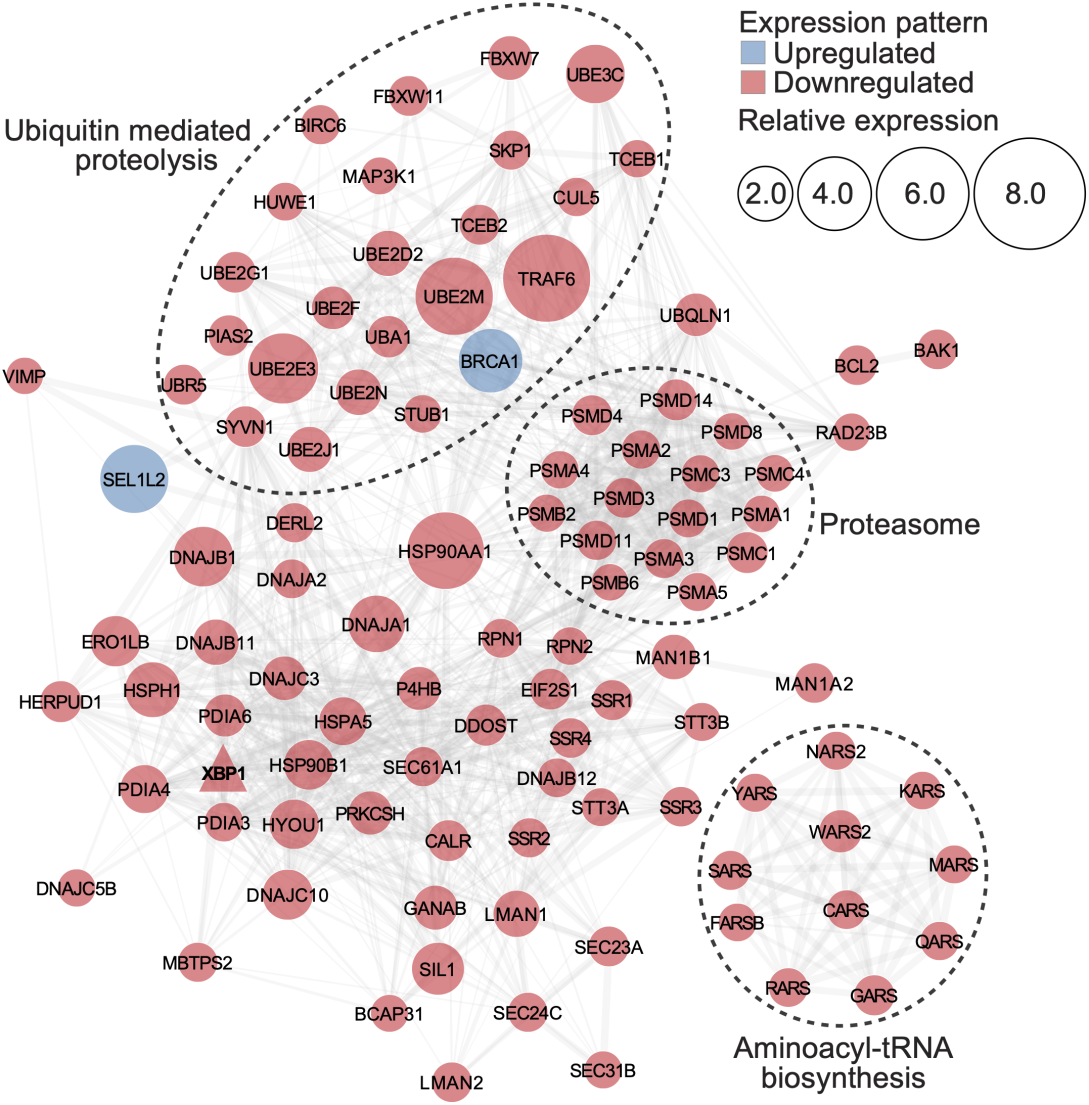
Unfolded protein response (UPR) interaction network. Node size indicates relative expression value computed as the sum of TPM values at RCP 8.5 relative to the Present Day samples. Node color represents pattern of expression (blue, downregulated; red, upregulated). Edge thickness denotes interaction confidence score. *XBP1* is shown as a triangular node. The network is based on human protein–protein interactions in the STRING database.

The interaction network of module L5 hub genes involved in protein folding, sorting, and degradation (hsa4141, hsa3050, and hsa4120), referred to hereafter as the “UPR interaction network”, is part of the adaptive or pro‐survival phase of the UPR (Figure 3). Upregulation of molecular chaperones, including heat shock protein family members (*HSP90B1*, *HSPA5*, *HSP90AA1*, and *HYOU1*), calreticulin (*CALR*), protein disulfide isomerases (*P4HB*, *PDIA6*), and B‐cell receptor‐associated protein (*BCAP31*), as well as co‐chaperones (*DNAJA2*, *DNAJB12*, *DNAJA1*, *DNAJB11*, *DNAJC10*, *DNAJC5B*, *DNAJC3*, *HSPH1*, and *SIL1*), may serve to enhance the capacity for protein folding (Walter & Ron, 2011). While generally linked to protein maturation, folding, structural maintenance, and transport, chaperones also play critical roles in eliminating aberrant proteins. For instance, *DNAJB12* and *DNAJB11* bind directly to unfolded proteins and divert them to ER‐associated protein degradation pathways (Jin et al., 2009).

N‐linked oligosaccharides attached to nascent proteins also function in protein folding and serve as tags for the selection of ER‐associated protein degradation substrates (Moremen & Molinari, 2006). Module L5 is enriched with genes involved in N‐glycan processing (GO6491) and protein glycosylation (GO6486) (Table S13). These include dolichyl‐diphosphooligosaccharide – protein glycosyltransferase subunits (*RPN1*, *RPN2*, *STT3A*, *STT3B*, and *DDOST*), which tag nascent polypeptide chains with a triglycosyl moiety, as well as glucosidases (*PRKCSH* and *GANAB*) (Figure 3), which convert triglycosylated to monoglycosylated proteins (Xu & Ng, 2015). Protein folding is facilitated by repeated interactions of monoglycosylated proteins with lectin chaperones such as *CALR* (Ruggiano et al., 2014; Tax et al., 2019), which also increased expression. *CALR*, along with other upregulated lectins (e.g., *LMAN1* and *LMAN2*), can recognize specific N‐linked oligosaccharide structures in improperly folded glycoproteins for degradation (Ruggiano et al., 2014; Tax et al., 2019). Activation of mannosyl‐oligosaccharide 1,2‐alpha‐mannosidase (*MAN1A2* and *MAN1B1*) and ER degradation‐enhancing alpha‐mannosidase‐like proteins (*EDEM1*, *EDEM2*, and *EDEM3*) (Table S14), which facilitate mannose trimming and substrate transfer to EDEM, signal the initiation of ER‐associated protein degradation mechanisms (Nishikawa et al., 2005; Oda et al., 2003).

Proteins triaged as terminally misfolded are retrotranslocated into the cytosol where they undergo a series of ubiquitination steps and are escorted into the proteasome for degradation (Smith et al., 2011). Enrichment of Ubiquitin‐Proteasome System (hsa4120 and hsa3050) genes in module L5 (Figure 2c), along with increased expression of translocation channel protein (*DERL2*) and its linker selenoprotein S (*VIMP*) (Figure 3), indicates active translocation and proteolysis of misfolded proteins. Module L5 contains ubiquitination complex components, including a ubiquitin‐activating enzyme E1 (*UBA1*), ubiquitin‐conjugating enzymes E2 (*UBE2M*, *UBE2G1*, *UBE2D2*, *UBE2J1*, *UBE2M*, *UBE2N*, *UBE2F*, *UBE2G1*, *UBE2D2*, *UBE2E3*, and *UBE2J1*), and ubiquitin ligase enzymes E3 (*STUB1*, *SYVN1*, *UBE3C*, *HUWE1*, *BIRC6*, *UBR5*, *PIAS2*, and *TRAF6*), which tag mutant proteins for degradation. Active degradation of polyubiquitinated substrates by the proteasome is evidenced by increased levels of the 20S (*PSMA1*, *PSMA2*, *PSMA3*, *PSMA4*, *PSMA5*, *PSMB2*, and *PSMB6*) and 26S (*PSMC1*, *PSMC3*, *PSMC4*, *PSMD1*, *PSMD3*, *PSMD4*, *PSMD8*, *PSMD11*, and *PSMD14*) proteasome subunits. *Leucetta chagosensis* may further extend its capacity to degrade misfolded proteins and damaged ER by mounting lysosome‐dependent pathways such as autophagy and mitophagy (Chipurupalli et al., 2021) (Figure 2c).

Another way by which the UPR relieves ER stress is by decreasing the flux of nascent proteins through repression of translational initiation (Pavitt & Ron, 2012). The UPR interaction network of *L. chagosensis* includes two homologs of *EIF2S1* (Figure 3), which can be converted by phosphorylated protein kinase‐R‐like ER kinase (*PERK*) into global protein synthesis inhibitors to facilitate translational attenuation during ER stress (Walter & Ron, 2011). However, previous studies have proposed that activation of biosynthetic pathways for specific amino acids is another adaptive mechanism to support protein synthesis demands of UPR‐activated genes (Gonen, Meller, et al., 2019; Gonen, Sabath, et al., 2019). In *L. chagosensis*, components of aminoacyl‐tRNA biosynthesis (hsa970) are enriched in module L5 (Figure 2c), along with other players involved in protein targeting and trafficking (i.e., translocon‐associated protein subunits (*SSR1*, *SSR1*, *SSR1*, and *SSR1*) and protein transport (*SEC23A*, *SEC24C*, *SEC31B*, and *SEC61A1*)), which suggests some degree of active translation. Module L5 also included 11 tRNA synthetases (Figure 3), five of which (*SARS*, *NARS2*, *QARS*, *CARS*, and *GARS*) load UPR‐induced amino acids (Ser, Asn, Glu, Cys, and Gly, respectively) on their cognate tRNAs (Gonen, Meller, et al., 2019).

GO enrichment analysis of module L5 revealed enrichment of genes linked to programmed cell death (GO12501) (Table S13). These include *CASP2*, an initiator protease essential for apoptosis execution (Vakifahmetoglu‐Norberg & Zhivotovsky, 2010), as well as both activators (*BAK1* and *SAP30BP*) and suppressors of apoptosis (*BCL2*, *TMBIM6*, and *BIRC6*) (Figure 3). The presence of apoptosis regulator and effector proteins in module L5 indicates that the UPR is in transition from the adaptive to the pro‐apoptotic phase.

### 3.5 Transcriptional regulators of the calcarean stress response

To identify key regulatory factors involved in the transcriptional stress response of *L. chagosensis*, we described the expression patterns of epigenetic modifiers and transcription factors in the sponge (Supplementary Results, Figure S7, Tables S15–S17). Some histone modifying enzymes, including acetyltransferases (*CBP* and *ELP*), deacetylases (*SIR2* and *SIR6*), methyltransferases (*KMT2E*, *SETB1*, and *EHMT1*), and demethylases (*KDM3B* and *KDM5A*), were classified as hub genes of module L5 (Table S18). Homologous genes such as *CBP‐1* in worm, *SIRT3* in mice, and *KDM4B* in human have all been shown to regulate the activation of UPR (Li et al., 2021; Wang et al., 2018; Xiaowei et al., 2023).

**FIGURE 4:**
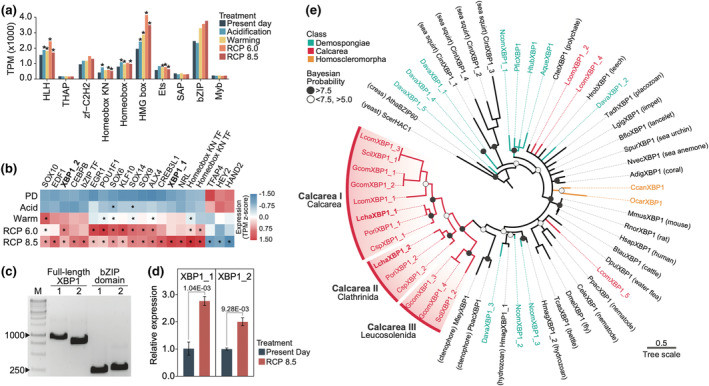
Stress‐responsive transcription factors in *Leucetta chagosensis*. (a) Bar plot showing the top 10 most abundant transcription factor families and their expression levels across treatments. Asterisks indicate significant Pfam enrichment (*p* < 0.05) relative to Present Day samples. (b) Differentially expressed transcription factors among module L5 hub genes. Expression levels (blue, low; red, high) are shown as TPM z‐score across all treatments. Asterisks indicate differential expression (FDR < 0.05) relative to the Present Day samples. PD, present day; Acid, acidification; Warm, warming. (c) Full‐length and bZIP domain amplicons of *LchaXBP1_1 and LchaXBP1_2*. M, 1 kb ladder. (d) Quantitation of *LchaXBP1* homologs in Present Day and RCP 8.5 samples. Statistical differences, computed using Student's *t*‐test, are indicated. (e) Phylogenetic analysis of *XBP1* homologs in sponges and other organisms. The phylogenetic tree was derived from Bayesian analysis of bZIP sequences (Data S1). Major calcarean *XBP1* clades are highlighted in red.

Several transcription factor families (i.e., Ets, bHLH, HMG box, and Homeobox KN) were also enriched in the set of upregulated genes at RCP 8.5 (Figure 4a, Table S12). Two of the most highly expressed families, HMG box and bZIP, showed a notable increase in expression under the tested conditions. To pinpoint key *trans*‐acting regulators involved in the UPR of *L. chagosensis*, putative transcription factors within the reconstructed UPR interaction network were identified. Module L5 includes 20 differentially expressed (FDR ≤ 0.05) transcription factors with high module membership (MM = 0.81–0.95; *p* = 4.89E‐07 – 8.39E‐04) (Figure 4b, Table S19). Of these 20 transcription factors, only *XBP1* is integrated into the UPR interaction network (Figure 3). *Leucetta chagosensis* has two homologs of *XBP1*: *LchaXBP1_1* (MM = 0.94; *p* = 1.15E‐06) and *LchaXBP1_2* (MM = 0.92; *p =* 7.15E‐06) and both were significantly upregulated at RCP 8.5 (Figure 4b–d).

### 3.6 *XBP1* homologs in *L. chagosensis* are lineage‐specific

Most metazoans possess a single copy of *XBP1* (Jindrich & Degnan, 2016), whereas calcareans, including *Clathrina* sp., *L. chagosensis*, *Pericharax orientalis*, *L. complicata*, *G. compressa*, and *Sycon ciliatum*, have at least two copies (Figure 4e, Table S7). Phylogenetic reconstruction indicates that calcarean *XBP1* genes are lineage‐specific, grouping into three major clusters, except for three *LcomXBP1* homologs (Figure 4e). *LchaXBP1_1* groups with other calcarean *XBP1 gene*s (*PoriXBP1_1*, *CspXBP1_1*, *LcomXBP1_1*, *GcomXBP1_1*, *GcomXBP1_2*, *ScilXBP1_1*, and *LcomXBP1_3* in the Calcarea I cluster), whereas *LchaXBP1_2* clusters with clathrinid *XBP1 gene*s (*PoriXBP1_2* and *CspXBP1_2* in the Calcarea II cluster). *XBP1* sequences from leucosolenids form a separate group (Calcarea III cluster). Homolog distribution suggests that *LchaXBP1_1* is the conserved ancestral paralog, whereas *LchaXBP1_2* is a derived copy that later diverged along with other clathrinid *XBP1* genes.

### 3.7 *LchaXBP1* homologs can form homo‐ and heterodimer pairs

**FIGURE 5:**
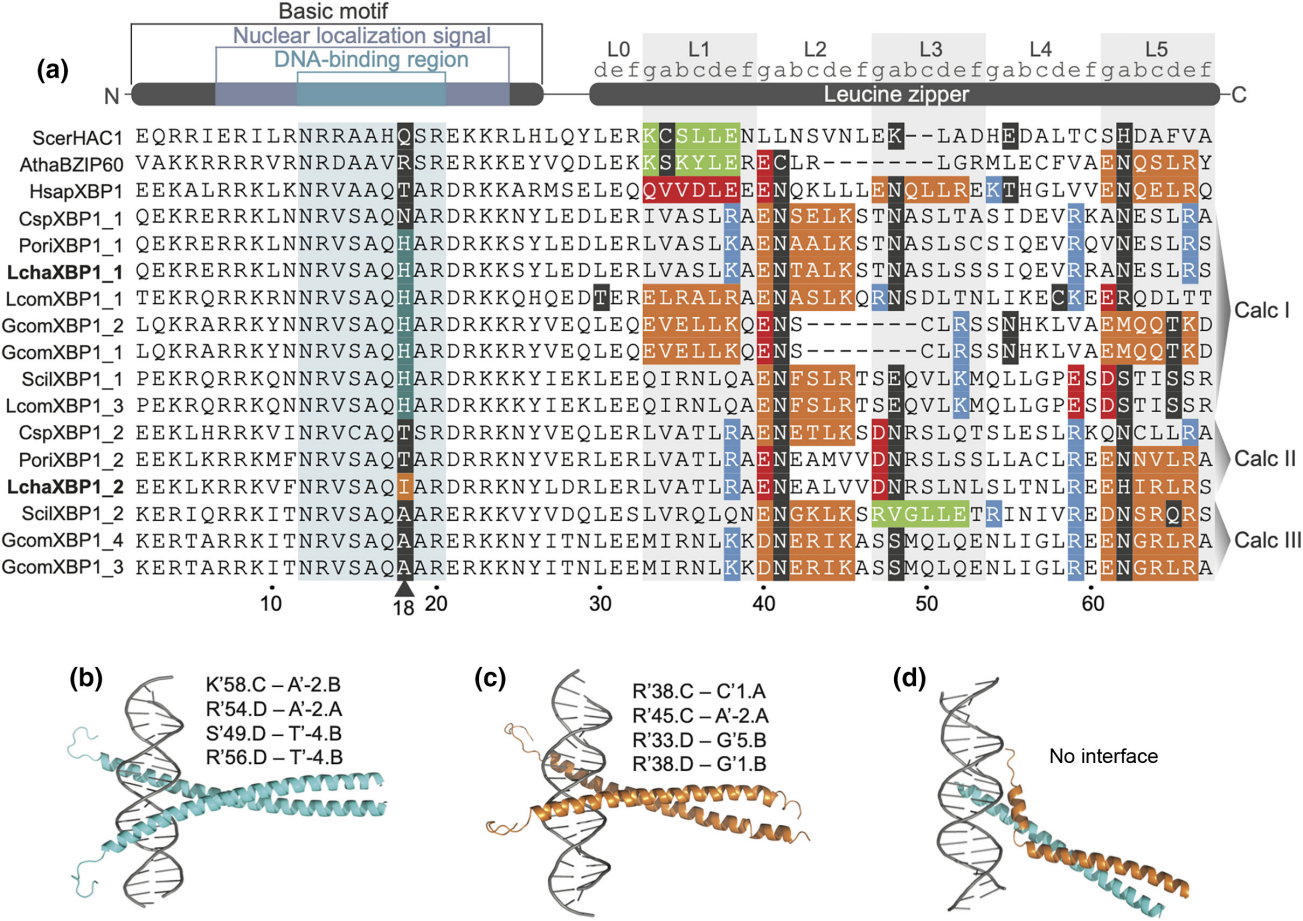
Dimerization and DNA‐binding potential of *XBP1* homologs. (a) Amino acid sequence alignment of the bZIP (PF00170) domain of *XBP1* homologs in yeast, cress, human, and Calcarea (Calc) I, II, and III clades. Residues in the L0‐L5 heptads of the leucine zipper domain are colored based on amino acid characteristics and potential interactions (green, attractive basic–acidic; orange, attractive acidic–basic; red, repulsive acidic pairs; blue, repulsive basic pairs; black, polar or charged amino acids). (b–d) Top predicted conformations for *LchaXBP1*‐CRE complexes. Structures of CRE with the (b) *LchaXBP1_1* (cyan) homodimer, (c) *LchaXBP1_2* (orange) homodimer, and (d) *LchaXBP1* heterodimer. All polar interactions between specific residues in *XBP1* and the cognate aureobox sequence within CRE are indicated for each complex.

Similar to other bZIP transcription factors, *XBP1* contains a bZIP domain with an N‐terminal DNA‐binding basic motif and C‐terminal dimerization leucine zipper domain (Hurst, 1995) (Figure 5a). A closer investigation of amino acid sequences revealed locus‐specific substitutions in the DNA‐binding region of *LchaXBP1* homologs (Figure 5a). Calcarea I members, including *LchaXBP1_1*, have a conserved His at position 18, whereas the Calcarea II member, *LchaXBP1_2*, has a species‐specific Ile at the same position. A previous study showed that His replacement in the DNA‐binding region changes the binding specificity of a bZIP transcription factor (Suckow et al., 1994). An Ile substitution, on the other hand, results in inhibition of DNA binding activity (Nantel & Quatrano, 1996). Since bZIPs need to dimerize in order to bind to dsDNA grooves (Bader & Vogt, 2006), it is hypothesized that different dimerization pairs of *LchaXBP1* homologs exhibit different DNA binding properties.

By comparing the structures of the leucine zipper heptads, we found that *LchaXBP1* homologs can form both homo‐ and heterodimer pairs through attractive interactions between acidic and basic amino acids at specific positions within the protein (Figure 5a, Supplementary Results). Homo‐ and heterodimer interaction signatures were also predicted for *XBP1* homologs in other clathrinids, *P. orientalis* and *Clathrina* sp. (Figure 5a).

### 3.8 *XBP1* homodimers but not the heterodimer bind to CRE

The structures of the *LchaXBP1_1* and *LchaXBP1_2* homodimers are similar to structures of the Transcription factor MafB (2wty.1.A) and Transcription factor MafG (3a5t.1.A) homodimers, respectively (Table S20). The structure of the *LchaXBP1* heterodimer is comparable to other bZIP heterodimers, such as the Transcription factor FosB/JunD (5vpd.2) pair. Based on in silico docking conformations (Table S21), it is predicted that *LchaXBP1* homodimers can bind to gene promoters through the cAMP responsive element (CRE), whereas the *LchaXBP1* heterodimer does not (Figure 5b–d). Indeed, *LchaXBP1* homodimers are predicted to form hydrogen bonds with specific nucleotides in the aureobox “TGACGT” within the CRE (Figure 5b,c, Supplementary Results, Table S22), which serves as the *cis*‐regulatory element for UPR activation (Clauss et al., 1996; Glimcher et al., 2020). In contrast, no polar interactions are predicted between the *LchaXBP1* heterodimer and CRE (Figure 5d, Supplementary Results, Table S22).

## 4 DISCUSSION

The current study is the first description of transcriptome‐wide changes accompanying the calcarean response to ocean acidification and warming. This work contributes to the limited sequence data resources for class Calcarea and, along with the bacterial community shifts and immune responses described in our previous study (Posadas et al., 2022), reveals a comprehensive view of gene regulatory networks underlying the stress response of the sponge holobiont.

*Leucetta chagosensis* activates components of the UPR under thermo‐acidic stress. The UPR detects misfolded proteins and enforces adaptive mechanisms by optimizing rates of protein synthesis and folding, as well as by initiating ER‐associated proteolytic pathways (Walter & Ron, 2011). Protein folding is a highly error‐prone process (Hebert & Molinari, 2007), which is aggravated under stressful conditions, such as high temperature and low pH, when the demands for protein folding exceed the capacity of the cell (Liu & Howell, 2010; Malhotra & Kaufman, 2007). Activation of the UPR has also been observed in the response of demosponges to elevated temperature stress. In particular, *S. officinalis* activates molecular chaperone genes, as well as other players involved in ER stress and UPR (Koutsouveli et al., 2020), while *H. tubifera* upregulates genes involved in protein refolding and proteolysis (Guzman & Conaco, 2016). Notably, *L. chagosensis* also activates the pro‐apoptotic phase of UPR, especially under the combination of extreme warming and acidification. Transition of the UPR from the pro‐survival to the pro‐apoptotic phase, which is mediated by the *PERK* (*via* *ATF4* and *CHOP* activities) or *IRE1*‐*CASP2* (*via* RIDD) pathways (Chen & Brandizzi, 2013; Walter & Ron, 2011), occurs when ER stress remains unmitigated because of sustained stress exposure. Switching between these two UPR phases requires strict regulatory mechanisms, which may determine survivorship under ER stress (Chan et al., 2015).

We identified two *XBP1* homologs, *LchaXBP1_1* and *LchaXBP1_2*, that may act as key regulators of the UPR network in *L. chagosensis*. Although most metazoans possess only a single copy of this gene (Jindrich & Degnan, 2016), a lineage‐specific duplication appears to have occurred in calcareous sponges. Indeed, phylogenetic reconstruction suggests that *LchaXBP1_1* is the conserved ancestral paralog, while *LchaXBP1_2* is a derived copy.

**FIGURE 6:**
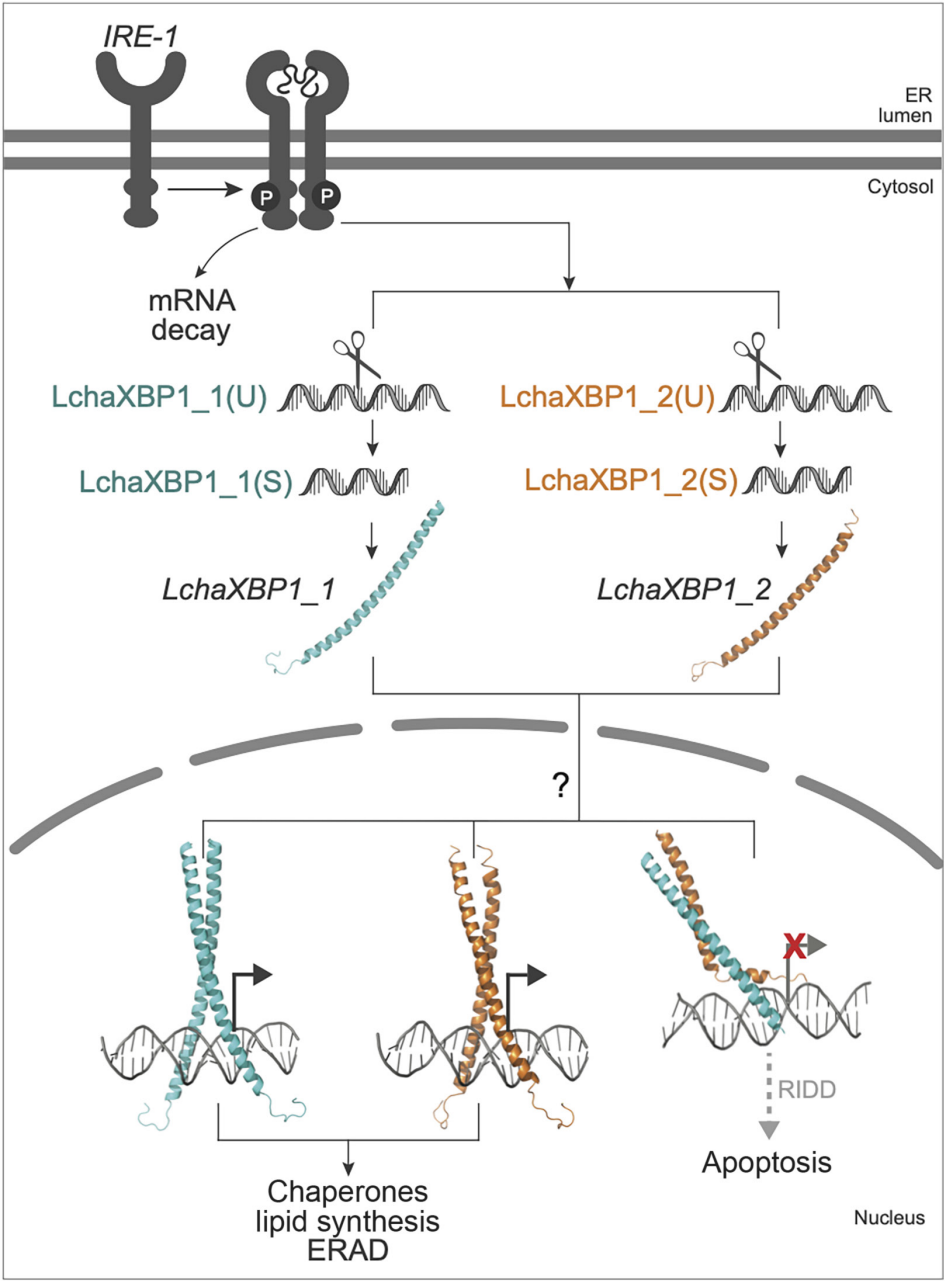
Proposed regulation of the UPR in *Leucetta chagosensis* through alternative dimerization of *LchaXBP1* homologs. Detection of unfolded proteins activates the endoribonuclease activity of *IRE‐1*, resulting in splicing of XBP1 mRNA (U). Translation of spliced XBP1 mRNA (S) produces peptides that can form dimers. *XBP1* homodimers bind to the CRE aureobox to activate pathways linked to the adaptive phase UPR. The *XBP1* heterodimer is unable to bind to the cognate DNA sequence, which allows activation of apoptosis through the RIDD pathway. ERAD, ER‐associated protein degradation.

Sequence analysis and structural modeling of the *L. chagosensis XBP1* genes reveal that these encode proteins that can combine to form dimers with differing abilities to bind to DNA. We propose that alternative dimerization of *XBP1* genes represents a mechanism to maintain the balance between adaptive and pro‐apoptotic phases of UPR in *L. chagosensis* (Figure 6). Specifically, homodimers of *XBP1* can bind to CRE and promote adaptive phase UPR, whereas heterodimer formation reversibly sequesters *LchaXBP1* into an inactive form that does not bind DNA, thereby permitting apoptosis through the RIDD pathway (Chan et al., 2015; Hetz et al., 2020). UPR regulation using this mechanism may be conserved among clathrinids since the *PoriXBP1* and *CspXBP1* heterodimers are also unable to bind to cognate DNA (Figure S8). Alternative dimerization of *bZIP* homo‐ and heterodimers has previously been described for *EmBP‐1* and *osZIP‐1a* transcription factors, with heterodimers losing the ability to bind to the Em1a DNA element to regulate gene expression in rice (Nantel & Quatrano, 1996).

Although the UPR is a highly conserved pathway from yeast to human (Wu et al., 2014), lineage‐specific duplication of *XBP1* in clathrinids may have resulted in a mechanism that enables rapid and strict regulation of pathways linked to the UPR. This mechanism is analogous to duplication of the stress‐responsive transcription factor, *MSN2–MSN4*, which resulted in adaptive gene expression tuning in yeast (Chapal et al., 2019). However, it should be noted that our results were inferred from in silico experiments and require further functional validation. Nonetheless, this demonstrates how transcription factor gene duplication promotes evolutionary innovation to further refine or expand the circuitry of transcription networks (Perez et al., 2014; Voordeckers et al., 2015). The upregulation of many paralogous genes in *L. chagosensis* (Figure S4) under thermo‐acidic stress also supports the idea that duplicated genes with roles in organismal stress response are typically selected for retention and exhibit stress‐specific transcriptional plasticity (Keane et al., 2014; Kuzmin et al., 2022; Mattenberger et al., 2017). Further characterization of other lineage‐ and species‐specific stress responsive genes in *L. chagosensis* may uncover a greater diversity of novel evolutionary solutions for maintaining organismal health in changing environments.

It is further interesting to note that, along with the components of UPR, genes and pathways that are associated with multiple neurodegenerative diseases are also enriched in the sponge under stress (Supplementary Results, Figure S9). Since ER stress is implicated in the development of several human pathologies (Lin et al., 2008; Marciniak & Ron, 2006), this coordinated activation may be further explored to point out ancestral molecular targets linking environmental etiologies to the development and progression of neurodegenerative diseases.

Population trajectory, breeding success, and known threats are some of the key factors that are typically used to assess species conservation status (Grace, 2023). Information derived from the transcriptional response of *L. chagosensis* to stress may provide additional tools for evaluating the susceptibility of calcareous sponges to different stressors. Molecular targets identified in this study can be leveraged in designing biomarker tools to investigate sublethal impacts that may contribute to population decline over generations. Our findings showing the susceptibility of *L. chagosensis* to future ocean conditions highlight the need to prioritize the conservation and management of calcisponges, which are often underappreciated in reef ecosystem studies.

## AUTHOR CONTRIBUTIONS

**Niño Posadas:** Conceptualization (equal); data curation (lead); formal analysis (lead); investigation (lead); methodology (equal); visualization (lead); writing – original draft (lead); writing – review and editing (equal). **Cecilia Conaco:** Conceptualization (equal); methodology (equal); funding acquisition (lead); project administration (lead); supervision (lead); writing – review and editing (equal).

## CONFLICT OF INTEREST STATEMENT

The authors declare no conflict of interest.

## Supporting information


Appendix S1:

## Data Availability

The datasets we generated and analyzed are available on Figshare (https://figshare.com/projects/Gene_regulatory_networks_in_calcareous_sponge_thermo‐acidic_stress_response/195164).
