# Supplementary material for: Gene networks governing the response of a calcareous sponge to future ocean conditions reveal lineage‐specific XBP1 regulation of the unfolded protein response
Source: Ecol Evol. 2024 Jun 30;14(7):e11652. doi: 10.1002/ece3.11652 (PMC11214833; doi:10.1002/ece3.11652)
Supplement: Supplementary file 1 — Appendix S1: [file ECE3-14-e11652-s001.pdf]

**Supplementary Information**

Gene networks governing the response of a calcareous sponge to future ocean conditions reveal lineage-specific *XBP1* regulation of the unfolded protein response

Niño Posadas and Cecilia Conaco


| **List of Contents** | **Page** |
|---|---|
| **Supplementary Methods** | 3-5 |
| **Supplementary Results** | 6-8 |
| **Supplementary Figures** | |
| **Fig. S1.** Sponge phylogeny and genes orthology | 9 |
| **Fig. S2.** Comparison of molecular functions | 10 |
| **Fig. S3.** Outline of $\alpha$-helices of two interacting leucine zippers | 11 |
| **Fig. S4.** Paralogs of activated genes | 11 |
| **Fig. S5.** Orthology of upregulated genes | 11 |
| **Fig. S6.** Eigengene expression of select gene modules | 12 |
| **Fig. S7.** Transcription factors in *Leucetta chagosensis* | 12 |
| **Fig. S8.** Binding potential of *XBP1* homologs | 13 |
| **Fig. S9.** Neurodegeneration-associated genes in module L5 | 14 |
| **Supplementary Tables** | |
| **Table S1.** Number of raw and cleaned sequencing reads | 15 |
| **Table S2.** Assembly statistics | 16 |
| **Table S3.** Annotation rate | 17 |
| **Table S4.** Sponge species included in comparative analyses | 18 |
| **Table S5.** Peptide sequences with detected homologs in other sponge species | 19 |
| **Table S6.** Distinguishing molecular functions in calcareous sponges | 20 |
| **Table S7.** *XBP1* homologs in sponges and other organisms | 21 |
| **Table S8.** *LchaXBP1* primers | 22 |
| **Table S9.** Gene modules in *Leucetta chagosensis* stress response | 22 |
| **Table S10.** Functional enrichment analysis for differentially expressed genes under RCP 8.5 | 23 |
| **Table S11.** Downregulated extracellular matrix components and biocalcification genes | 24 |
| **Table S12.** Pfam enrichment analysis for up- and downregulated genes | 25 |
| **Table S13.** Functional enrichment analysis for modules L1, L4, and L5 | 26 |
| **Table S14.** Expression pattern of EDEM proteins across all treatments | 27 |

| List of Contents | Page |
|---|---|
| **Table S15.** Histone acetylation proteins in *Leucetta chagosensis* | 28 |
| **Table S16.** Histone methylation proteins in *Leucetta chagosensis* | 29 |
| **Table S17.** DNA methylation machinery components in *Leucetta chagosensis* | 30 |
| **Table S18.** Epigenetic modifiers in module L5 hub genes | 30 |
| **Table S19.** Transcription factors in module L5 hub genes | 31 |
| **Table S20.** Structural homologs of *XBP1* dimer pairs | 32 |
| **Table S21.** Top docking conformation for *XBP1*-CRE complexes | 33 |
| **Table S22.** Binding potential of *XBP1* dimers to CRE | 34 |
| **Supplementary Data** | |
| **Data S1.** Aligned and trimmed amino acid sequences of bZIP domain in *XBP1* homologs in sponges and other organisms | 35-40 |
| **Literature cited** | 41-44 |

## Supplementary Methods

Generating a non-redundant transcriptome for *Leucetta chagosensis*

Raw sequence reads were trimmed using Trimmomatic v0.32 (1). Poor-quality bases (quality score < 3) at the start and end of the reads, as well as the first 15 bases from the start of the reads, were removed. Reads were also trimmed if the average per-base quality within a 4-base sliding window fell below 20 and if the length is < 36 bases. *De novo* transcriptome assembly was carried out using Trinity (2). Transcripts with 90% sequence similarity were clustered and the longest representative contigs (> 300bp) were retained. Isoforms with the highest combined IsoPct or longest length were retained for each transcript to generate a non-redundant reference transcriptome. Isoforms with zero isoform percentage (IsoPct) were removed. Quality and completeness of the assemblies were assessed using Bowtie 2 v2.3.5.1 (3), Detonate (4), TransRate v1.0.3 (5), and BUSCO v3.1.0 (6). The reference transcriptome was then annotated by aligning against the UniProtKB/Swiss-Prot database (April 2020) with an e-value cut-off of $1 \times 10^{-5}$.

Peptides were predicted using the Transdecoder package in Trinity and annotated by alignment against the GenBank non-redundant (nr) sequence and UniProtKB/Swiss-Prot databases with an e-value cut-off of $1 \times 10^{-5}$. The top Blastp hit for each peptide was used as input into OmicsBox (BioBam, Valencia, Spain) (7) to predict gene ontology (GO) annotations. Protein domains were identified by mapping the peptide sequences against Pfam 32.0 database (8) using HMMER v3.3 (9). The top Blastx and Blastp hits in UniProtKB/Swiss-Prot database and Pfam annotations for each transcript were then used as input into Trinotate v3.2.2 (10) to generate a comprehensive annotation report. GO annotations generated in OmicsBox and Trinotate were merged to improve the annotation rate.

Sequence similarity, ortholog analysis, and gene content comparison

Pairwise sequence comparisons between *L. chagosensis* and other sponge species were performed using Blastp alignments at an e-value cutoff of $1 \times 10^{-5}$. A total of 18 sponge species representing the four poriferan classes were included in the analysis (Table S4). These include demosponges (*Amphimedon queenslandica* Hooper & van Soest, 2006 (11), *H. tubifera* (12), *Petrosia (Petrosia) ficiformis* (Poiret, 1789) (13), *X. testudinaria*, *S. carteri* (14), *Aplysina aerophoba* (Nardo, 1833), *Dysidea avara* (Schmidt, 1862) (15), *Ephydatia muelleri* (Lieberkühn, 1856) (16), *Neopetrosia compacta* (Ridley & Dendy, 1886) (17)), calcareans (*Sycon ciliatum*, *L. complicata* (Montagu, 1814) (18), *Grantia compressa* (Fibricius, 1780), *Pericharax orientalis* van Soest & De Voogd, 2015, *Clathrina sp.* (19)), homoscleromorphs (*Oscarella carmela* Muricy & Pearse, 2004, *Corticum candelabrum* Schmidt, 1862), and a hexactinellid (*Aphrocallistes vastus* (Schulze, 1886)).

Predicted peptide sequences of representative sponge species were annotated against the Pfam 32.0 (8) database and were assigned to their associated GO terms based on Blastp top hits (e-value $\leq 1 \times 10^{-5}$) in UniProtKB/Swiss-Prot database. Differences in the distribution of peptides that are assigned to GO terms among sponges were visualized through PCA. Percent abundance was computed relative to the total number of predicted peptides in each species. Molecular functions that distinguish calcareans from other sponges (LDA score ≥ 2, $p < 0.05$) were determined using Linear Discriminant Analysis effect size (LDA-LEfSe) (20).

Orthologous gene families in the transcriptome of *L. chagosensis* and in the genomes or transcriptomes of other sponge species were identified using OrthoFinder (21). Intersections of orthologous groups across different species were visualized using the UpSetR package in R (22).

## Identification of gene regulatory elements in *L. chagosensis*

Transcription factors (23), as well as epigenetic modifiers, including histone modifying enzymes (24-27) and components of DNA methylation machinery (28), were identified in the *L. chagosensis* transcriptome based on their characteristic domains and top Blastp hit (e-value < $1 \times 10^{-5}$) against the UniProtKB/Swiss-Prot database.

## Differential gene expression analysis

Transcript abundance was estimated by mapping reads to the reference transcriptomes using RNA-Seq by Expectation Maximization (29) with bowtie2 alignment (3). DEGs were identified using the edgeR (30) package in R. Generalized linear model functionality for likelihood ratio testing method, which is recommended for datasets with few replicates (30), was applied. Expected counts were converted to counts per million (CPM) and only genes (n = 22 417) with > 2 CPM in at least two libraries were included in edgeR analysis. This filtering step was done to remove lowly expressed genes (< 10 counts). Genes were considered differentially expressed if upregulation or downregulation was ≥ 4-fold relative to the controls with a False Discovery Rate (FDR) ≤ 0.05. Pairwise comparisons were conducted between the Present Day samples and samples subjected to the other treatments.

## Sequencing and quantitation of *LchaXBP1* homologs

Total RNA was extracted from the tissues of sponge individuals exposed to Present Day control and RCP 8.5 treatments (n=3 per treatment) using TRIzol reagent (Invitrogen, Waltham, MA, USA). Contaminating DNA was removed using the TURBO DNA-free Kit (Invitrogen). cDNA synthesis was carried out using the GoScript Reverse Transcriptase kit (Promega, Madison, WI, USA). Full-length and bZIP domains of the two *XBP1* genes in *L. chagosensis* were amplified using specific primers (Table S8). Amplicons were sent

to Macrogen, South Korea, for Sanger sequencing. Trimmed and aligned sequences were deposited in GenBank under the accession numbers: PP716769 (LcXBP1_1), PP627506 (LcXBP1_2), PP627507 (LcbZIP2650), and PP627508 (LcbZIP60042).

Expression levels of the two *XBP1* homologs in *L. chagosensis* under the Present Day and RCP 8.5 treatments were estimated by quantitative PCR on a QuantStudio 3 Real-Time PCR system (Thermo Fisher Scientific). Quantitative PCR reactions included 40 cycles of activation at 95°C for 2 min, denaturation at 95°C for 15 sec, and annealing/elongation at 55°C for 1 min. Each reaction contained 10 µl of 2X GoTaq qPCR Master Mix (Promega), 0.2 µl each of 100 µM forward and reverse LcbZIP primers, 4 µl template cDNA, and 5.6 µl nuclease-free $H_2O$. Three biological replicates and three technical replicates were used in the quantitation of each gene alongside negative controls. Primer efficiency and primer specificity were assessed using dilution curves and melt curves, respectively. The abundance of target transcripts was computed using the Pfaffl method (31). Target transcript abundances were normalized to β-tubulin expression. Statistical differences were calculated using the paired two-sample Student's t-Test.

## Identification of *XBP1* dimer pairs

Leucine zipper heptads of *LchaXBP1, PoriXBP1,* and *CspXBP1* homologs were manually evaluated to identify possible dimerization pairs. Heptads (L0-L5) were grouped (*gabcdef*) to visualize amino acids in the *a*, *d*, *e*, and *g* positions, which regulate dimerization stability and specificity of bZIP transcription factors (Fig. S3). The complementary *a* ↔ *a'* and *d* ↔ *d'* interactions create a hydrophobic core that is essential for dimer stability (32) while electrostatic interactions between *g* ↔ *e'* pair can either be attractive or repulsive (33). Attractive basic-acidic interactions include Arg ↔ Glu and Lys ↔ Glu while Glu ↔ Arg, Glu ↔ Lys, Asp ↔ Arg, and Asp ↔ Lys are attractive acidic-basic interactions. Glu ↔ Glu, Glu ↔ Asp, Glu ↔ Gln, and Gln ↔ Glu form acidic repulsive interactions, whereas basic repulsive interactions include Lys ↔ Lys, Arg ↔ Lys, Gln ↔ Lys, Arg ↔ Gln, and Lys ↔ Gln (34).

## Supplementary Results

Generating a reference transcriptome for *L. chagosensis*

We sequenced the transcriptome of *L. chagosensis* on the NovaSeq 6000 platform, generating an average of 19 299 672 clean 100 bp paired-end reads (Table S1). *De novo* transcriptome assembly rendered 248 731 total transcripts. The non-redundant transcriptome, following filtering through isoform selection and sequence clustering, is composed of 91 886 (N50 = 1 409; Ex90N50 = 2 463) transcripts (Table S2). The largest contig is 46 605 bp while the smallest contig is 300 bp long. The transcriptome has 45.94% GC content, 99.20% of all bases are covered by reads, and 87.29% of reads mapped back to the assembly. Assembled transcripts were translated into 44 538 peptides (Table S3). Ortholog benchmarking indicates that the transcriptome contains 93.40% and 90.80% of the eukaryotic and metazoan core genes, respectively (Table S2).

Gene repertoire of *Leucetta chagosensis*

The gene repertoire of *L. chagosensis* is comparable to other calcareous sponges (Table S5) with 538 orthogroups (904 *L. chagosensis* genes) present among calcareans, and 412 (1 433 *L. chagosensis* genes) and 455 (2 332 *L. chagosensis* genes) orthologous gene sets shared among clathrinids and leucettids, respectively (Fig. S1). 1 401 putative species-specific genes, assigned to 205 orthogroups, were also identified in the assembly.

Comparison of GO annotations showed high similarity of the *L. chagosensis* functional repertoire to other calcareous sponges (Fig. S2A). LDA-LEfSe revealed that calcareans are relatively enriched for genes involved in G protein-coupled receptor activity (GO4930), extracellular matrix structural constituent (GO5201), calcium ion binding (GO5509), and transcriptional control (i.e., chromatin binding (GO3682), DNA binding (GO978), and DNA binding transcription factor activity (GO981)), among other functions (Fig. S2B, Table S6).

Gene regulatory elements in *Leucetta chagosensis*

An extensive complement of putative histone modifiers involved in acetylation (histone acetyltransferases (HATs) and histone deacetylases (HDACs)) and methylation (histone methyltransferases (HMTs) and histone demethylases (HDMs)) were detected in the transcriptome of *L. chagosensis* (Table S15-16). Core components of the DNA methylation machinery were also identified in the assembly (Table S17).

A diverse repertoire of transcription factor genes (n = 505) were detected in *L. chagosensis*. The most abundant are *bHLH* (n = 81), *THAP* (n = 53), *C2H2-type zinc finger,* (n = 48), *Homeobox KN* (n = 41), Homeobox (n = 40), *HMG box* (n = 30), *Ets* (n = 26), *SAP* (n = 19), *bZIP* (n = 17), and *Myb* (n = 17) (Fig. S7). The great diversity of

transcription factor genes in *L. chagosensis* corroborates findings from genome-wide surveys of developmental transcription factors in the calcisponge, *S. ciliatum*, which revealed expanded families and presence of Calcarea-specific groups of *bHLH*, *Homeobox*, and *HMG box* (18, 35, 36).

## Predicted dimerization of *XBP1* homologs

Attractive acidic - basic *g* ↔ *e'* pairs are found in the *LchaXBP1_1* homodimer (2nd heptad), *LchaXBP1_2* homodimer (5th heptad), and *LchaXBP1* heterodimer (2nd and 5th heptads) (Fig. 5A). *LchaXBP1* homologs also contain Asn in position *a* of the 2nd, 3rd, and 5th heptads, which likely promotes both homo- and heterodimerization (33). Other heterodimerizing leucine zippers comprising any combination of the three aliphatic amino acids: Ile (e.g., *LchaXBP1_1*, 4th heptad), Leu (e.g., *LchaXBP1_2*, 4th heptad), and Val (e.g., 1st heptad) in the *a* position have similar coupling energies (34). The prevalence of Leu in the *d* position also contribute to dimer stability due to the unique packing interactions of the two Leu and their neighboring amino acids (33).

## Predicted binding of *XBP1* to CRE

In the *LchaXBP1_1* homodimer bound to CRE (Fig. 5B), residue Arg'42 (C/NH2) forms hydrogen bonds with nucleotides dG'-6(A/O6) and dG'5(B/O6), Ser'49 (D/OG) with dT'-4(B/O4), Arg'54 (D/NH1) with dA'-2(A/O5'), Arg'56 (C/NH2; D/NH2) with dG'-6(A/O3) and dT'-4(B/O3'), and Lys'58 (C/NZ) with DA'-2(B/O3') (Table S22). In the *LchaXBP1_2* homodimer bound to CRE (Fig. 5C), residue Arg'33 (D/NH2) forms hydrogen bonds with nucleotide dG'5 (B/O6), Arg'38 (C/NE; D/NH2) with dC'-1(A/O3') and dG'1 (B/O3'), Arg'45 (C/NE; D/NE) with dA'-2(A/O5') and dA'-2.B/O3', and Arg'47 (C/NH2) with dC'-5 (B/O3') (Table S22).

## Neurodegeneration genes are co-expressed with UPR components

Endoplasmic reticulum stress is implicated in the development of several human pathologies, including diabetes mellitus, obesity, cardiovascular diseases, cancer, and neurodegenerative disorders (37, 38). Age-related neurodegeneration is often linked to loss of potency, as well as alterations in adaptive UPR signaling (39). Intriguingly, we found that along with the components of UPR, genes and pathways that are associated with multiple neurodegenerative diseases are enriched in module L5 (Fig. S9A). These include amyotrophic lateral sclerosis (hsa5014), Huntington disease (hsa5016), spinocerebellar ataxia (hsa5017), Parkinson disease (hsa5012), Alzheimer disease (hsa5010), and prion disease (hsa5020). Some of the member genes have been described as part of the evolutionarily conserved neural degeneration pathways in colonial tunicates (e.g., *GNAQ*, *PLCB4*, *NOTCH1*, *CYCS*, *BECN1*, *CSNK2B*, *CREBBP*, *RELN*, *CTNNB1*, *GAPDH*, *SLC18A2*, *INSR*, *LRP5*, *CDK5*, and *IDE*) (Fig. S9B) (40).

Although sponges do not possess any structure resembling a functional neuron (41), they have an almost complete set of gene homologs found in mammalian synapses (11). The detection of neurodegeneration-related genes in *L. chagosensis* can be compared to the observed overlap of core multicellularity genes in the genome of *A. queenslandica* with the set of genes that are implicated in cancer (11). This highlights the possibility that genomic events linked to early animal evolution can be informative about ancestrally conserved mechanisms that drive aberrant functioning of metazoan-specific traits. The coordinated activation of neurodegeneration-associated genes with UPR components in *L. chagosensis* under stress may be further elucidated to point out ancestral molecular targets linking environmental etiologies to the development and progression of neurodegenerative diseases.

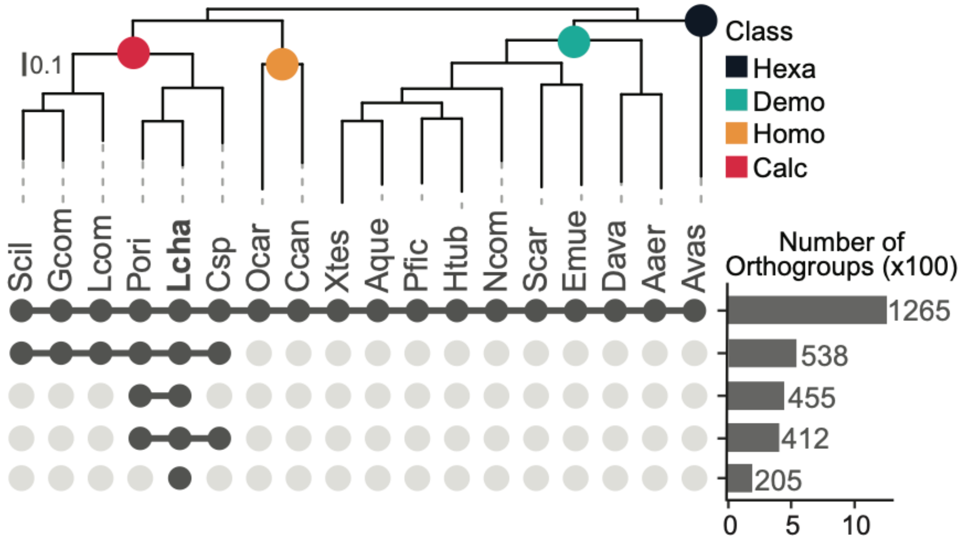


**Fig. S1. Sponge phylogeny and gene orthology.** The bar graph represents the number of orthogroups that are common amongst the sponge species indicated above the dark circles. Only orthogroups with at least 4 gene members from any species are shown. The species tree was inferred from all genes by the STAG algorithm and rooted using STRIDE (21). Species abbreviations: *Sycon ciliatum* (Scil), *Grantia compressa* (Gcom), *Leucosolenia complicata* (Lcom), *Pericharax orientalis* (Pori), *Leucetta chagosensis* (Lcha), *Clathrina* sp. (Csp), *Oscarella carmela* (Ocar), *Corticum candelabrum* (Ccan), *Xestopongia testudinaria* (Xtes), *Amphimedon queenslandica* (Aque), *Petrosia ficiformis* (Pfic), *Haliclona tubifera* (Htub), *Neopetrosia compacta* (Ncom), *Stylissa carteri* (Scar), *Ephydatia muelleri* (Emue), *Dysidea avara* (Dava), *Aplysina aerophoba* (Aaer), *Aphrocallistes vastus* (Avas).

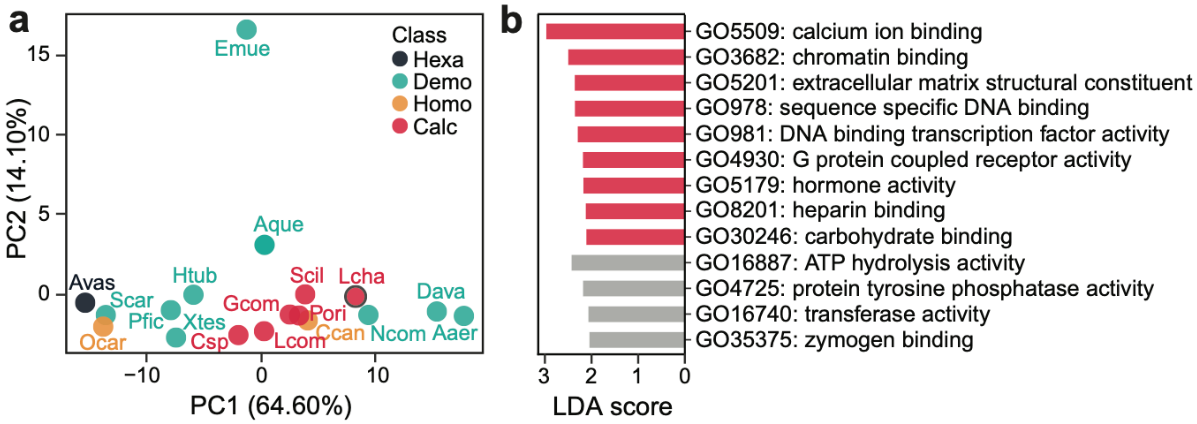


**Fig. S2. Comparison of the gene repertoire of *Leucetta chagosensis* with other sponge species. (a)** Principal component analysis was based on the relative abundance of peptides with matches to Molecular Functions in the Gene Ontology database for all species. Relative abundance was computed as the percentage of peptides associated with a function relative to the total number of predicted peptides in each species. **(b)** Distinguishing molecular functions in calcareous sponges. Functions that distinguish (LDA score > 2; *p*-value <0.05) between calcareans (red) and other sponge species (grey) were determined using LDA-LEfSe based on relative abundance values. Species abbreviations: *Sycon ciliatum* (Scil), *Grantia compressa* (Gcom), *Leucosolenia complicata* (Lcom), *Pericharax orientalis* (Pori), *Leucetta chagosensis* (Lcha), *Clathrina* sp. (Csp), *Oscarella carmela* (Ocar), *Corticum candelabrum* (Ccan), *Xestopongia testudinaria* (Xtes), *Amphimedon queenslandica* (Aque), *Petrosia ficiformis* (Pfic), *Haliclona tubifera* (Htub), *Neopetrosia compacta* (Ncom), *Stylissa carteri* (Scar), *Ephydatia muelleri* (Emue), *Dysidea avara* (Dava), *Aplysina aerophoba* (Aaer), *Aphrocallistes vastus* (Avas).

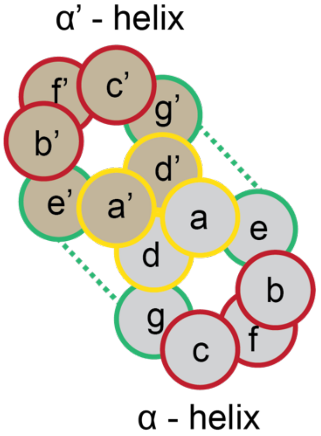


**Fig. S3. Outline of α-helices of two interacting leucine zippers.** Amino acids in positions *a* and *d* configure the hydrophobic core (yellow). Charged residues in positions *e* and *g* generate electrostatic forces (green dashed lines). The hydrophilic surface is formed by the amino acids in positions *b*, *c*, and *f* (red).

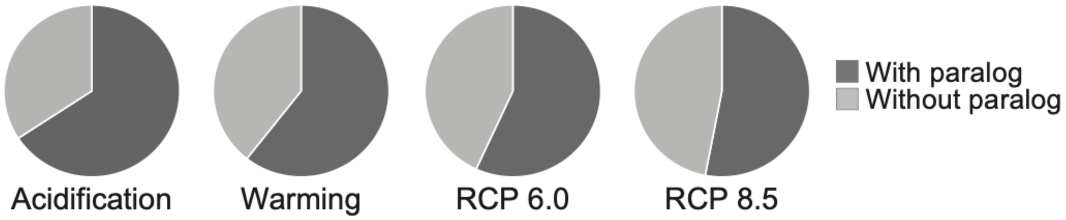


**Fig. S4. Paralogs of stress-responsive genes in *L. chagosensis*.** Percent of upregulated genes with (dark grey) and without (light grey) detectable paralogs. Detection of paralogs was based on OrthoFinder (21) output.

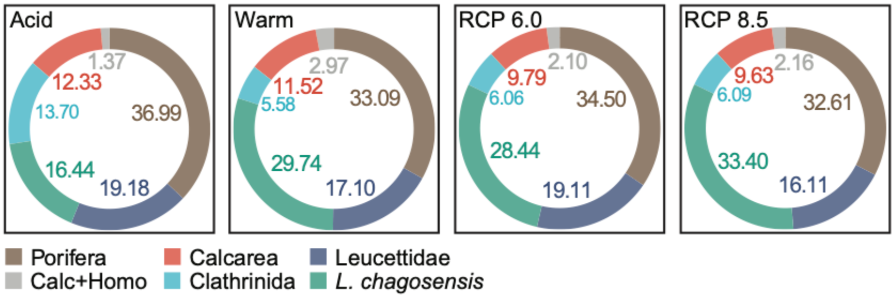


**Fig. S5. Orthology of upregulated genes.** Upregulated genes were classified based on their orthogroup type. Numbers indicate percent of genes in each orthogroup type relative to the total upregulated genes for each treatment. Colors denote different lineages. Assignment of orthogroup types was based on OrthoFinder (21) output.

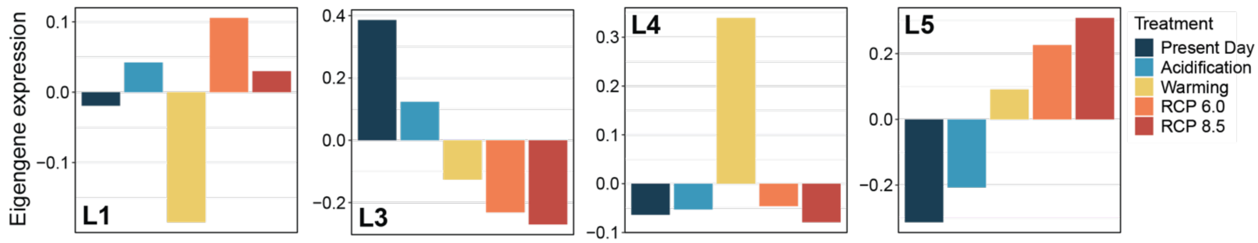


**Fig. S6. Eigengene expression representing select gene modules.** Modules that are positively correlated with the four treatment conditions (module L1 = RCP 6.0, module L3 = Present Day, module L4 = Warming, module L5 = RCP 8.5) are shown. Colors indicate different treatments.

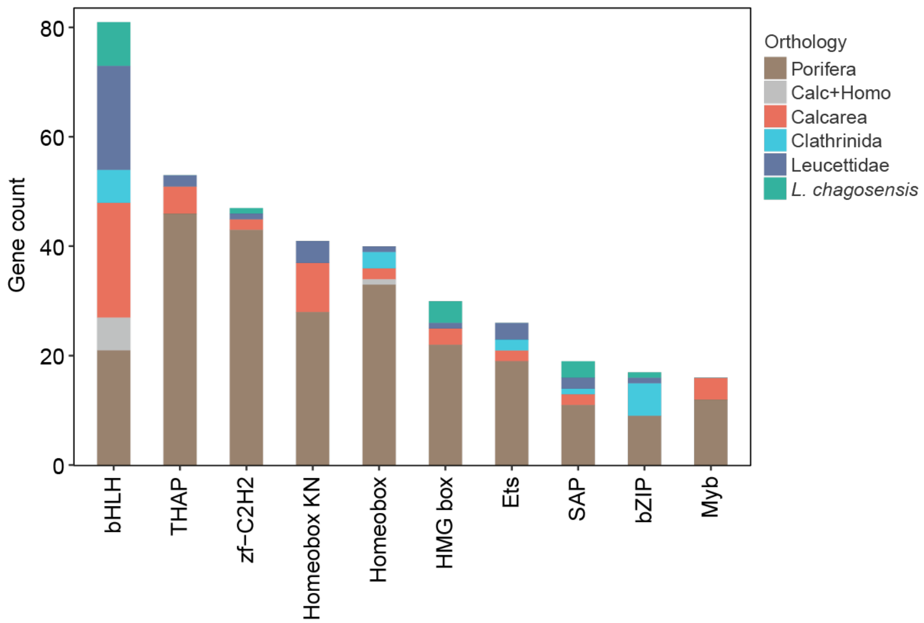


**Fig. S7. Transcription factors in *Leucetta chagosensis*.** Bar plot shows counts and orthology assignments of the top 10 most abundant transcription factor families. Orthology assignments were based on OrthoFinder (21) output.

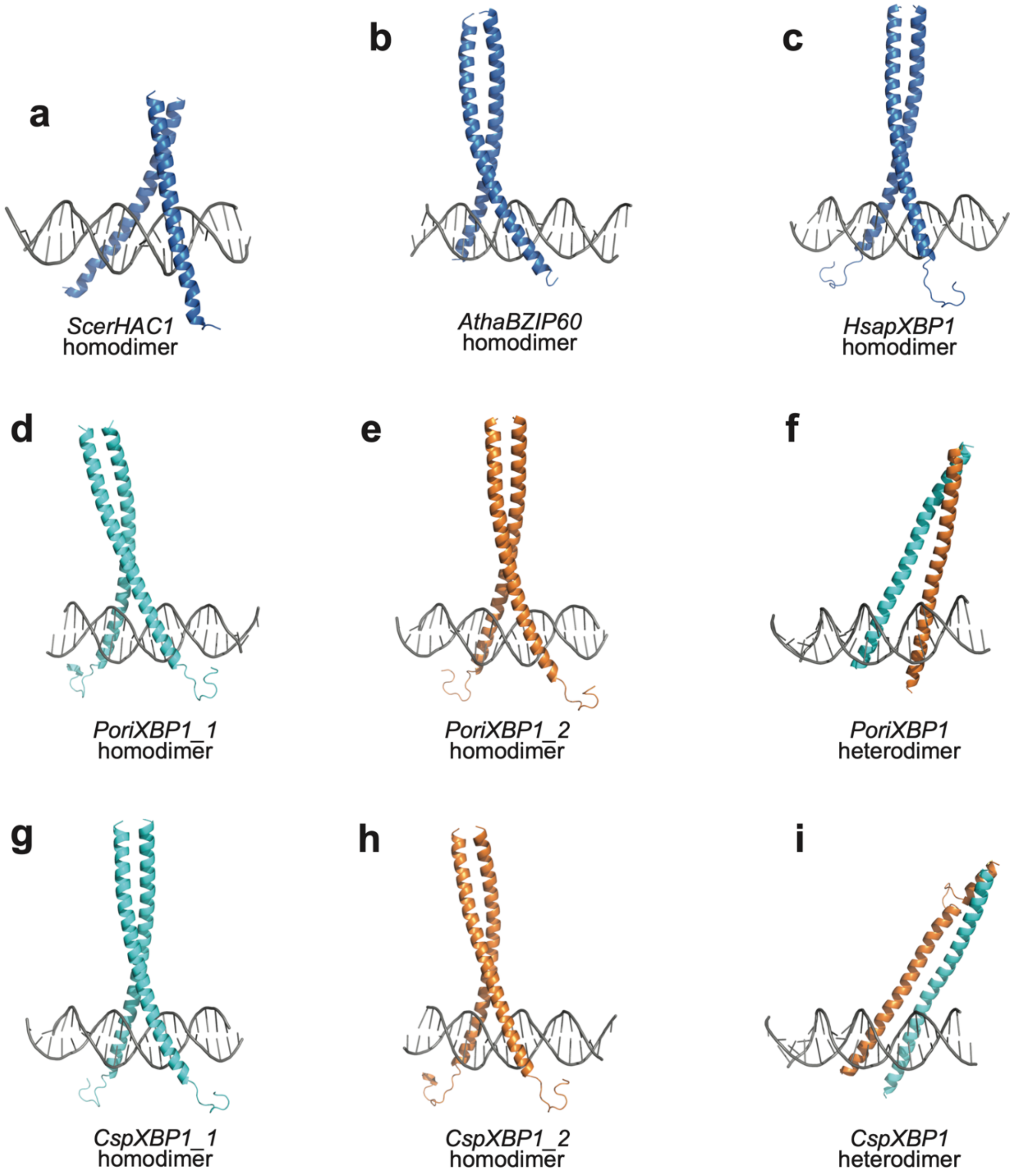


**Fig. S8. DNA-binding potential of *XBP1* homologs.** Predicted conformations of CRE with dimers of **(a)** *HAC1* in yeast, **(b)** *bZIP60* in plant, **(c)** *XBP1* in human, **(d-f)** *PorXBP1,* and **(g-i)** *CspXBP1*. Dimers generated in the docking experiment were rendered in Pymol (15). *XBP1* homologs are denoted by chain colors (cyan, *XBP1_1*; orange, *XBP1_2*).

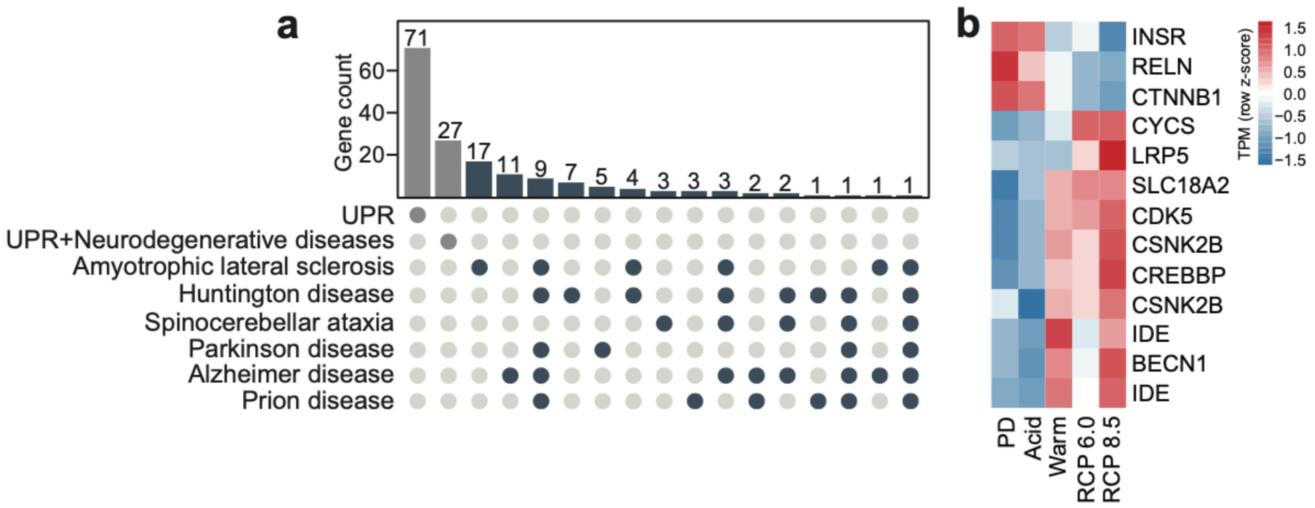


**Fig. S9. Neurodegeneration-associated genes in module L5. (a)** Enriched pathways linked to the UPR and neurodegenerative diseases. The bar graph represents the number of genes that are common amongst the KEGG pathways listed on the left. Genes that are exclusively associated with the UPR or linked to both the UPR and neurodegenerative diseases are colored grey. Genes that are exclusive to neurodegenerative diseases are colored blue. **(b)** Module L5 genes that are part of evolutionarily-conserved neural degeneration pathways (40). Expression levels of each gene are shown as TPM z-score across all treatments (low, blue; high, red).

**Table S1.** Number of raw and cleaned reads generated from transcriptome sequencing of *Leucetta chagosensis* under the different treatments.

| | | Number of reads | | |
|---|---|---|---|---|
| Treatment /Sample | Library | Before trimming | After trimming | Retained (%) |
| Lcha_PD_1 | BL6 | 18 667 724 | 18 120 773 | 97.07 |
| Lcha_PD_2 | DL10 | 17 514 288 | 16 898 940 | 96.49 |
| Lcha_PD_3 | FL6 | 18 933 592 | 18 269 880 | 96.49 |
| Lcha_Acid_1 | BL3 | 21 394 219 | 20 765 512 | 97.06 |
| Lcha_Acid_2 | DL3 | 21 668 998 | 20 966 926 | 96.76 |
| Lcha_Acid_3 | FL3 | 21 362 844 | 20 714 129 | 96.96 |
| Lcha_Warm_1 | BL10 | 20 717 841 | 20 063 796 | 96.84 |
| Lcha_Warm_2 | FL10 | 19 215 770 | 18 667 193 | 97.15 |
| Lcha_RCP6.0_1 | BL9 | 19 669 045 | 18 986 196 | 96.53 |
| Lcha_RCP6.0_2 | IH1 | 21 938 576 | 21 239 062 | 96.81 |
| Lcha_RCP8.5_1 | BL5 | 20 207 469 | 19 522 256 | 96.61 |
| Lcha_RCP8.5_2 | DL5 | 15 753 886 | 15 279 402 | 96.99 |
| Lcha_RCP8.5_3 | FL5 | 21 982 086 | 21 401 675 | 97.36 |
| | | average | 19 299 672 | 96.86 |

**Table S2.** Assembly statistics of the *Leucetta chagosensis* transcriptome. Statistics at major filtering steps are shown.

| | |
|---|---|
| **Trinity** | |
| Number of genes | 158 138 |
| Number of transcripts | 248 731 |
| GC (%) | 46.38 |
| N50 | 1 258 |
| **Isoform selection** | |
| Number of genes | 93 355 |
| Number of transcripts | 93 355 |
| GC (%) | 45.95 |
| N50 | 1 396 |
| **Sequence clustering (90% similarity)** | |
| Number of genes | 91 886 |
| Number of transcripts | 91 886 |
| GC (%) | 45.94 |
| N50 | 1 409 |
| Ex90N50 | 2 463 |
| Smallest contig (bp) | 300 |
| Largest contig (bp) | 46 605 |
| Bases covered by reads (%) | 99.20 |
| Alignment rate (%) | 87.29 |
| Mean orf (%) | 59.54 |
| BUSCO Eukaryota odb9 (%) | 93.40 |
| BUSCO Metazoa odb9 (%) | 90.80 |
| BUSCO Alveolata odb10 (%) | 88.30 |
| BUSCO Bacteria odb9 (%) | 32.40 |

**Table S3.** Number of predicted peptides and annotation rate of the *Leucetta chagosensis* transcriptome.

| | |
|---|---|
| **Number of peptides** | 44 538 |
| Genbank nr (nr) | 3 190 |
| UniProt | 35 |
| Pfam | 753 |
| GO | 813 |
| nr + UniProt | 36 |
| nr + Pfam | 884 |
| nr + GO | 263 |
| UniProt + Pfam | 17 |
| UniProt + GO | 413 |
| Pfam + GO | 1 052 |
| nr + UniProt + Pfam + GO | 13 188 |
| nr + UniProt + Pfam | 150 |
| nr + UniProt + GO | 1 416 |
| UniProt + Pfam + GO | 1 419 |
| nr + Pfam + GO | 553 |
| **Annotated peptides** | 24 182 (54.30%) |

**Table S4.** Sponge species included in comparative analyses. LMA, Low Microbial Abundance; HMA, High Microbial Abundance. Sources of the predicted peptide sequences are indicated.

| Species | Class | Subclass | Host type | Source |
|---|---|---|---|---|
| *A. vastus* | Hexactinellida | Hexasterophora | LMA | Ana Riesgo[a] |
| *A. aerophoba* | Demospongiae | Verongimorpha | HMA | Lucia Pita Galan[b] |
| *D. avara* | Demospongiae | Keratosa | LMA | Lucia Pita Galan[b] |
| *E. muelleri* | Demospongiae | Heteroscleromorpha | | EphyBase[c] |
| *S. carteri* | Demospongiae | Heteroscleromorpha | LMA | Compagen[d] |
| *N. compacta* | Demospongiae | Heteroscleromorpha | HMA | Genbank[e] |
| *H. tubifera* | Demospongiae | Heteroscleromorpha | LMA | Compagen[d] |
| *P. ficiformis* | Demospongiae | Heteroscleromorpha | HMA | Ana Riesgo[a] |
| *A. queenslandica* | Demospongiae | Heteroscleromorpha | LMA | Ensembl Metazoa[f] |
| *X. testudinaria* | Demospongiae | Heteroscleromorpha | HMA | Compagen[d] |
| *C. candelabrum* | Homoscleromorpha | NA | HMA | Ana Riesgo[a] |
| *O. carmela* | Homoscleromorpha | NA | LMA | Compagen[d] |
| *Clathrina* sp. | Calcarea | Calcinea | LMA | LMU Open data repository[g] |
| *L. chagosensis* | Calcarea | Calcinea | LMA | This study |
| *P. orientalis* | Calcarea | Calcinea | LMA | LMU Open data repository[g] |
| *L. complicata* | Calcarea | Calcaronea | LMA | Compagen[d] |
| *G. compressa* | Calcarea | Calcaronea | LMA | LMU Open data repository[g] |
| *S. ciliatum* | Calcarea | Calcaronea | LMA | Compagen[d] |

a, Ana Riesgo (Museo Nacional de Ciencias Naturales, Madrid, Spain)
b, Lucia Pita Galan (Institute of Marine Science, Barcelona, Spain)
c, EphyBase (spaces.facsci.ualberta.ca/ephybase/)
d, Compagen (42)
e, Genbank (ncbi.nlm.nih.gov/nuccore/GIYW00000000)
f, Ensembl Metazoa (metazoa.ensembl.org)
g, LMU Open data repository (data.ub.uni-muenchen.de/202/)

**Table S5.** Number of *Leucetta chagosensis* peptides with homologs (blastP e-value $\leq 1 \times 10^{-5}$) in other sponge species.

| Species | Class | Number of peptides (%) | Average Identity (%) |
|---|---|---|---|
| *A. vastus* | Hexactinellida | 29.71 | 38.44 |
| *A. aerophoba* | Demospongiae | 39.77 | 38.63 |
| *D. avara* | Demospongiae | 43.93 | 39.32 |
| *E. muelleri* | Demospongiae | 40.47 | 39.67 |
| *S. carteri* | Demospongiae | 38.03 | 40.52 |
| *N. compacta* | Demospongiae | 41.54 | 40.82 |
| *H. tubifera* | Demospongiae | 38.74 | 39.06 |
| *P. ficiformis* | Demospongiae | 38.114 | 39.89 |
| *A. queenslandica* | Demospongiae | 41.36 | 39.98 |
| *X. testudinaria* | Demospongiae | 34.04 | 39.97 |
| *C. candelabrum* | Homoscleromorpha | 39.95 | 41.52 |
| *O. carmela* | Homoscleromorpha | 33.63 | 40.5 |
| *Clathrina sp.* | Calcarea | 47.89 | 51.85 |
| *P. orientalis* | Calcarea | 61.37 | 61.86 |
| *L. complicata* | Calcarea | 52.91 | 45.24 |
| *G. compressa* | Calcarea | 49.42 | 44.35 |
| *S. ciliatum* | Calcarea | 51.14 | 44.92 |

**Table S6.** Distinguishing molecular functions in calcareous sponges. Functions that distinguish (LDA score > 2; *p*-value <0.05) between calcareans and other sponge species were determined using LDA-LEfSe based on relative abundance values. Abundance was computed as percentage of peptides associated with a specific molecular function relative to the total number of predicted peptides in each species.

| Function | LDA score | p-value | Relative abundance (%) | | |
|---|---|---|---|---|---|
| | | | Lcha | Calcarea (ave) | Others (ave) |
| GO30246: carbohydrate binding | 2.12 | 2.00E-03 | 0.48 | 0.56 | 0.39 |
| GO3682: chromatin binding | 2.50 | 2.73E-03 | 1.10 | 1.21 | 0.92 |
| GO4930: G protein-coupled receptor activity | 2.19 | 3.12E-02 | 0.48 | 0.57 | 0.41 |
| GO5179: hormone activity | 2.18 | 1.05E-03 | 0.23 | 0.24 | 0.05 |
| GO5201: extracellular matrix structural constituent | 2.36 | 6.61E-03 | 0.53 | 0.67 | 0.36 |
| GO5509: calcium ion binding | 2.98 | 3.69E-03 | 2.80 | 3.37 | 2.25 |
| GO8201: heparin binding | 2.13 | 6.61E-03 | 0.33 | 0.43 | 0.27 |
| GO978: RNA polymerase II cis-regulatory region sequence-specific DNA binding | 2.36 | 1.14E-02 | 0.90 | 0.88 | 0.68 |
| GO981: DNA-binding transcription factor activity, RNA polymerase II-specific | 2.30 | 3.12E-02 | 0.96 | 0.84 | 0.68 |
| GO16740: transferase activity | 2.07 | 1.14E-02 | 0.22 | 0.19 | 0.40 |
| GO16887: ATP hydrolysis activity | 2.43 | 3.94E-02 | 0.99 | 1.44 | 1.97 |
| GO35375: zymogen binding | 2.05 | 2.73E-03 | 0.04 | 0.02 | 0.20 |
| GO4725: protein tyrosine phosphatase activity | 2.19 | 3.69E-03 | 0.25 | 0.31 | 0.57 |

**Table S7.** List of *XBP1* homologs in sponges and other organisms. Asterisks indicate sequences from Jindrich and Degnan, 2016 (43). Sources of the predicted peptide sequences are indicated.

| Species | *XBP1* homologs |
|---|---|
| *Saccharomyces cerevisiae* (yeast)[a] | sp\|P41546\|HAC1_YEAST |
| *Arabidopsis thaliana* (plant)[a] | sp\|Q9C7S0\|BZP60_ARATH |
| *Mnemiopsis leydii* (ctenophore) | ML47553a (NHGRI: Mnemio_ML2.2.aa)* |
| *Pleurobrachia bachei* (ctenophore) | sb\|3462249\| (Neurobase)* |
| *Dysidea avara*[b] | m.207939, m.318648, m.366491, m.73633, m.89309 |
| *Neopetrosia compacta*[c] | m.38196, m.45707, m.25476 |
| *Haliclona tubifera*[d] | mm.4791 |
| *Petrosia ficiformis*[e] | m.6984 |
| *Amphimedon queenslandica* | 15722756 (Ensembl Metazoa)* |
| *Corticium candelabrum*[e] | m.34032 |
| *Oscarella carmela* | g8106.t1 (Compagen)* |
| *Clathrina* sp.[f] | m.20007, m.25898 |
| *Leucetta chagosensis* | m.10477, m.8707 |
| *Pericharax orientalis*[f] | m.34365, m.75641 |
| *Leucosolenia complicata*[d] | lcpid114286\|, lcpid149412\|, lcpid197212\|, lcpid81423\|, lcpid148212\| |
| *Grantia compressa*[f] | m.20320, m.20321, m.127570, m.127572 |
| *Sycon ciliatum* | scpid77518\|, scpid80538\|* |
| *Trichoplax adhaerans* (placozoan) | 64159 (jgi\|Triad1\|)* |
| *Acropora digitifera* (coral) | adi_v1.04236 (OIST)* |
| *Nematostella vectensis* (sea anemone) | 156374297 (NCBI)* |
| *Hydra magnipapillata* (freshwater polyp) | 221091242 (NCBI), 221123354 (NCBI)* |
| *Helobdella robusta* (leech)[g] | HelroP193172 |
| *Capitella telata* (polychaete) | 221349 (jgi\|Capca1\|)* |
| *Lottia gigantea* (limpet) | 232105 (jgi\|Lotgi1\|)* |
| *Daphnia pulex* (water flea) | 314438 (jgi\|Dappu1\|)* |
| *Tribolium castaneum* (red flour beetle) | 91091398 (ncbi)* |
| *Drosophila melanogaster* (fruit fly) | FBpp0289469 (Ensembl)* |
| *Pristionchus pacificus* (nematode)[g] | PPA15410 |
| *Caenorhabditis elegans* (nematode)[a] | sp\|G5EE07\|XBP1_CAEEL |
| *Strongylocentrotus purpuratus* (sea urchin) | 72085815 (NCBI)* |
| *Ciona intestinalis* (sea squirt) | ENSCINP00000024434, ENSCINP00000010446, ENSCINP00000021189, ENSCINP00000015310 (Ensembl)* |
| *Branchiostoma floridae* (lancelet) | 260833700 (Ensembl)* |
| *Rattus norvegicus* (rat)[a] | sp\|Q9R1S4\|XBP1_RAT |
| *Mus musculus* (mouse)[a] | sp\|O35426\|XBP1_MOUSE |
| *Bos taurus* (cattle)[a] | sp\|Q3SZZ2\|XBP1_BOVIN |
| *Homo sapiens* (human) | 14110395 (NCBI)* |

a, UniProtKB/Swiss-Prot database
b, Lucia Pita Galan (Institute of Marine Science, Barcelona, Spain)
c, Genbank (ncbi.nlm.nih.gov/nuccore/GIYW00000000)
d, Compagen (42)
e, Ana Riesgo (Museo Nacional de Ciencias Naturales, Madrid, Spain)
f, LMU Open data repository (data.ub.uni-muenchen.de/202/)
g, Ensembl Metazoa (metazoa.ensembl.org)

**Table S8.** List of primer pairs used for sequencing and quantitation of *LchaXBP1* homologs.

| Gene | Primers |
|---|---|
| LchaXBP1_1 | Fwd: 5'-GTTCGTGTTGTAGTTGTAGAGTAG-3'<br>Rev: 5'- TTATTTGGCCGCATTCATTTACAC -3' |
| LchaXBP1_2 | Fwd: 5'-CATTAGCCAGAGCGTTGGATTGTC-3'<br>Rev: 5'-AGATCCCTTCATCCTCTTCCGAATTC-3' |
| LchabZIP_1 | Fwd: 5'-CGAGAAGCTTGACCACCTGT-3'<br>Rev: 5'-TCTGTCTTACCCCTCCTTGC-3' |
| LchabZIP_2 | Fwd: 5'-CCTGAAAAGTATCGGCCGAG-3'<br>Rev: 5'-GTGAGGCTGGCAATGTTGAA-3' |
| Lchaβtubulin | Fwd: 5'-AAGCTCACAACACCGACCTA-3'<br>Rev: 5'-TGCTGAGATCCACGACTTGT-3' |

**Table S9.** Gene modules in *Leucetta chagosensis* stress response. Hub genes (GS ≥ \|0.2\|; MM> \|0.8\|) were identified for modules that are positively correlated with the four treatments (module L1 = RCP 6.0, module L3 = Present Day, module L4 = Warming, module L5 = RCP 8.5). Hub genes were annotated through Blastp search (e-value < $1x10^{-5}$) against the human proteome v 11.5 from STRING v.11 database (44). GS, gene significance; MM, module membership.

| Module | Number of genes | Hub genes | Hub genes with STRINGdb hits |
|---|---|---|---|
| L1 | 5 282 | 316 | 111 (35.13%) |
| L2 | 1 509 | - | - |
| L3 | 7 582 | 2 577 | 1 584 (61.46%) |
| L4 | 2 121 | 398 | 176 (44.22%) |
| L5 | 5 803 | 1 849 | 1 346 (72.08%) |

**Table S10.** Functional enrichment analysis for differentially expressed genes ($log_2$ Fold change > |2|; FDR < 0.05) under RCP 8.5. GO terms with *p*-value < 0.05 are considered significantly enriched. Enriched terms were summarized through REVIGO (45) at 0.5 similarity cut-off.

| GO ID | Term | Number of genes | topgoFisher (*p*-value) |
|---|---|---|---|
| GO:0006351 | DNA-templated transcription | 87 | 1.52E-03 |
| GO:0035329 | hippo signaling | 9 | 7.30E-04 |
| GO:0030154 | cell differentiation | 105 | 2.41E-03 |
| GO:0016477 | cell migration | 40 | 9.00E-03 |
| GO:0007160 | cell-matrix adhesion | 12 | 6.40E-04 |
| GO:0030198 | extracellular matrix organization | 15 | 1.61E-02 |
| GO:0032963 | collagen metabolic process | 7 | 3.51E-02 |
| GO:0045669 | positive regulation of osteoblast differentiation | 6 | 3.73E-02 |
| GO:0008152 | metabolic process | 293 | 1.75E-02 |
| GO:0051924 | regulation of calcium ion transport | 5 | 5.35E-03 |
| GO:0005975 | carbohydrate metabolic process | 31 | 1.56E-03 |
| GO:0006730 | one-carbon metabolic process | 6 | 2.31E-02 |
| GO:0016042 | lipid catabolic process | 11 | 4.00E-05 |
| GO:0009607 | response to biotic stimulus | 37 | 3.30E-04 |
| GO:0071229 | cellular response to acid chemical | 12 | 1.40E-05 |
| GO:0030162 | regulation of proteolysis | 16 | 6.38E-03 |
| GO:0050790 | regulation of catalytic activity | 44 | 4.42E-03 |
| GO:0006955 | immune response | 35 | 2.60E-08 |
| GO:0007166 | cell surface receptor signaling pathway | 95 | 2.90E-04 |
| GO:0002224 | Toll-like receptor signaling pathway | 10 | 7.54E-03 |
| GO:0033209 | TNF-mediated signaling pathway | 9 | 5.80E-03 |
| GO:0042981 | regulation of apoptotic process | 40 | 1.45E-02 |

**Table S11.** Downregulated ($log_2$ Fold change < -2; FDR < 0.05) extracellular matrix components and biocalcification genes at RCP 8.5.

| Transcript | Peptide | Fold change (log2) | UniProt top blastP hit (e-value ≤1 × $10^{-5}$) |
|---|---|---|---|
| TRINITY_DN870_c0_g1_i1 | m.60353 | -2.34 | CO1A2_CANLF (collagen alpha-2(I) chain) |
| TRINITY_DN20179_c0_g1_i1 | m.13564 | -2.69 | CO1A2_RAT (collagen alpha-2(I) chain) |
| TRINITY_DN3760_c0_g2_i1 | m.17803 | -2.23 | CO1A2_RAT (collagen alpha-2(I) chain) |
| TRINITY_DN20145_c0_g1_i7 | m.13526 | -2.09 | CO1A2_RAT (collagen alpha-2(I) chain) |
| TRINITY_DN6310_c0_g1_i1 | m.15091 | -3.11 | CO4A1_DROME (collagen alpha-1(IV) chain) |
| TRINITY_DN4873_c0_g1_i1 | m.57604 | -3.06 | CO6A5_MOUSE (collagen alpha-5(VI) chain) |
| TRINITY_DN870_c0_g2_i1 | m.60356 | -2.38 | COLL2_MIMIV (collagen-like protein 2) |
| TRINITY_DN2692_c0_g1_i1 | m.8689 | -2.41 | COLL5_MIMIV (collagen-like protein 5) |
| TRINITY_DN311_c0_g1_i2 | m.5744 | -5.14 | CTHR1_HUMAN (collagen triple helix repeat-containing protein 1) |
| TRINITY_DN66893_c0_g3_i2 | m.18184 | -2.74 | ITB6_HUMAN (integrin beta-6) |
| TRINITY_DN17023_c0_g1_i2 | m.15768 | -4.20 | FBN2_HUMAN (fibrillin-2) |
| TRINITY_DN2687_c0_g2_i2 | m.8838 | -3.25 | FBN3_HUMAN (fibrillin-3) |
| TRINITY_DN1447_c1_g1_i3 | m.14748 | -3.57 | MLP_ACRMI (mucin-like protein) |
| TRINITY_DN6916_c0_g1_i1 | m.19609 | -2.67 | MUC5B_HUMAN (mucin-5B) |
| TRINITY_DN394_c0_g1_i5 | m.5633 | -3.43 | CAH1_GORGO (carbonic anhydrase 1) |
| TRINITY_DN80101_c0_g1_i1 | m.52914 | -4.28 | CAH2_RABIT (carbonic anhydrase 2) |
| TRINITY_DN95379_c0_g1_i1 | m.63095 | -3.17 | CAH2_RABIT (carbonic anhydrase 2) |
| TRINITY_DN78691_c0_g1_i2 | m.9678 | -3.81 | CAH7_HUMAN (carbonic anhydrase 7) |

**Table S12.** Pfam enrichment analysis for up- and downregulated genes ($log_2$ Fold change > |2|; FDR < 0.05) across all treatments. Pfam domains with *p*-value < 0.05 are considered significantly enriched.

| Expression pattern | Pfam | Number of genes (enrichment p-value) | | | |
|---|---|---|---|---|---|
| | | Acidification | Warming | RCP 6.0 | RCP 8.5 |
| Up | Ank | | 9 (5.00E-03) | 11 (6.00E-03) | 11 (2.00E-02) |
| Up | CUB | | 4 (4.00E-04) | 4 (1.00E-03) | 5 (8.00E-05) |
| Up | DAN | | | 3 (3.00E-02) | 3 (3.00E-02) |
| Up | EGF | | | | 14 (6.00E-06) |
| Up | EGF_CA | | | | 15 (8.00E-05) |
| Up | Ets | | 3 (2.00E-02) | 5 (1.00E-04) | 4 (6.00E-03) |
| Up | HLH | 3 (4.00E-02) | 4 (3.00E-02) | 10 (3.00E-08) | 6 (4.00E-03) |
| Up | HMG_box | 3 (3.00E-03 | 4 (1.00E-03) | 6 (5.00E-06) | 4 (7.00E-03) |
| Up | Homeobox | 4 (9.00E-04) | 5 (1.00E-03) | | 5 (1.00E-02) |
| Up | Homeobox_KN | 3 (7.00E-03) | | 5 (1.00E-03) | 4 (3.00E-02) |
| Up | Lipoxygenase | | 5 (8.00E-06) | 6 (4.00E-07) | 5 (8.00E-05) |
| Up | PB1 | | 4 (1.00E-04) | 3 (1.00E-02) | 4 (5.00E-04) |
| Up | UBA_5 | | 2 (5.00E-03) | | 2 (1.00E-02) |
| Up | zf-C3HC4_4 | | | | 3 (5.00E-02) |
| Up | zf-TRAF | | | | 6 (1.00E-03) |
| Down | 7tm_2 | | | | 11 (2.00E-06) |
| Down | ATP-gua_Ptrans | | 2 (4.00E-02) | 3 (1.00E-03) | 2 (2.00E-02) |
| Down | Band_3_cyto | | 2 (2.00E-02) | 2 (4.00E-02) | |
| Down | Cadherin | | | 5 (2.00E-03) | 7 (3.00E-04) |
| Down | Carb_anhydrase | | | | 4 (6.00E-03) |
| Down | COLFI | | 5 (1.00E-07) | | 5 (3.00E-05) |
| Down | Collagen | | 7 (2.00E-08) | 5 (6.00E-04) | 11 (1.00E-10) |
| Down | DAN | | 4 (7.00E-05) | | 4 (3.00E-03) |
| Down | DERM | | | | 5 (9.00E-06) |
| Down | EGF | | 6 (2.00E-02) | 12 (6.00E-06) | 16 (2.00E-06) |
| Down | Ependymin | | 3 (5.00E-04) | 3 (2.00E-03) | 5 (2.00E-06) |
| Down | HCO3_cotransp | | 2 (2.00E-02) | 2 (3.00E-02) | |
| Down | PID | | | 3 (3.00E-02) | 4 (7.00E-03) |
| Down | Pkinase | | | | 17 (3.00E-03) |
| Down | Sulfatase | | | 7 (3.00E-04) | 10 (1.00E-05) |
| Down | TNF | | | | 3 (7.00E-03) |
| Down | TSP_1 | | 4 (2.00E-02) | 5 (1.00E-02) | 8 (3.00E-04) |

**Table S13.** Functional enrichment analysis for modules L1, L4, and L5 components. Terms with *p*-value < 0.01 were considered significantly enriched.

| Module | GO ID | Term | Number of genes | topgoFisher (p-value) |
|---|---|---|---|---|
| **L1** | GO:0007205 | protein kinase C-activating G protein-coupled receptor signaling pathway | 10 | 1.52E-03 |
| | GO:0043552 | positive regulation of phosphatidylinositol 3-kinase activity | 16 | 5.40E-03 |
| | GO:0051781 | positive regulation of cell division | 10 | 9.90E-04 |
| | GO:0007009 | plasma membrane organization | 57 | 4.57E-03 |
| | GO:0042157 | lipoprotein metabolic process | 34 | 3.31E-03 |
| | GO:0034767 | positive regulation of monoatomic ion transmembrane transport | 21 | 8.14E-03 |
| | GO:0051592 | response to calcium ion | 17 | 1.07E-03 |
| | GO:0006412 | translation | 125 | 4.30E-07 |
| | GO:0072659 | protein localization to plasma membrane | 41 | 3.14E-03 |
| | GO:0007166 | cell surface receptor signaling pathway | 298 | 1.27E-03 |
| | GO:0035872 | nucleotide-binding domain, leucine rich repeat containing receptor signaling pathway | 16 | 9.01E-03 |
| | GO:1901224 | positive regulation of NIK/NF-kappaB signaling | 17 | 2.68E-03 |
| | GO:0051092 | positive regulation of NF-kappaB transcription factor activity | 32 | 9.55E-03 |
| | GO:0032760 | positive regulation of tumor necrosis factor production | 14 | 4.98E-03 |
| **L4** | GO:2000045 | regulation of G1/S transition of mitotic cell cycle | 10 | 5.57E-03 |
| | GO:0044260 | cellular macromolecule metabolic process | 431 | 8.55E-03 |
| | GO:0006468 | protein phosphorylation | 105 | 4.52E-03 |
| | GO:0051865 | protein autoubiquitination | 16 | 6.26E-03 |
| | GO:0006955 | immune response | 83 | 7.64E-03 |
| **L5** | GO:0070830 | bicellular tight junction assembly | 10 | 6.06E-03 |
| | GO:0007173 | epidermal growth factor receptor signaling pathway | 36 | 1.33E-03 |
| | GO:0006996 | organelle organization | 635 | 2.25E-03 |
| | GO:0044772 | mitotic cell cycle phase transition | 86 | 8.27E-03 |
| | GO:0030859 | polarized epithelial cell differentiation | 11 | 4.58E-03 |
| | GO:0002053 | positive regulation of mesenchymal cell proliferation | 11 | 3.00E-03 |
| | GO:0010975 | regulation of neuron projection development | 74 | 8.95E-03 |
| | GO:0001501 | skeletal system development | 56 | 6.20E-03 |
| | GO:0044260 | cellular macromolecule metabolic process | 1 627 | 4.80E-04 |
| | GO:0008299 | isoprenoid biosynthetic process | 11 | 9.39E-03 |
| | GO:0045892 | regulation of DNA-templated transcription | 132 | 5.10E-04 |
| | GO:0006397 | mRNA processing | 104 | 4.80E-04 |
| | GO:0043488 | regulation of mRNA stability | 20 | 5.10E-04 |
| | GO:0006457 | protein folding | 79 | 1.40E-04 |
| | GO:0006486 | protein glycosylation | 86 | 9.46E-03 |
| | GO:0006491 | N-glycan processing | 11 | 6.80E-05 |
| | GO:0051291 | protein heterooligomerization | 19 | 1.26E-03 |
| | GO:0015031 | protein transport | 410 | 1.90E-04 |
| | GO:0050714 | positive regulation of protein secretion | 33 | 4.62E-03 |
| | GO:0030433 | ubiquitin-dependent ERAD pathway | 23 | 1.11E-03 |
| | GO:0038095 | Fc-epsilon receptor signaling pathway | 23 | 5.90E-03 |
| | GO:0016236 | macroautophagy | 47 | 9.29E-03 |
| | GO:0012501 | programmed cell death | 333 | 2.14E-03 |
| | GO:0050691 | regulation of defense response to virus by host | 18 | 4.00E-04 |

**Table S14.** Expression pattern of EDEM transcripts across all treatments. Transcript counts are presented as TPM (mean ± sd). Annotations were based on blastP search (e-value < $1x10^{-5}$) against the human proteome v 11.5 from STRING v.11 database (44). PD, Present Day; Acid, Acidification; Warm, Warming.

| Transcript | Peptide | Annotation | Expression level (TPM) | | | | |
|---|---|---|---|---|---|---|---|
| | | | PD | Acid | Warm | RCP 6.0 | RCP 8.5 |
| TRINITY_DN14284_c0_g2_i9 | m.27098 | EDEM1 9606.ENSP00000256497 | 11.83 ±0.52 | 11.23 ±3.49 | 14.01 ±2.26 | 12.64 ±0.73 | 15.34 ±1.53 |
| TRINITY_DN6389_c0_g1_i2 | m.15269 | EDEM2 9606.ENSP00000363616 | 10.88 ±0.70 | 10.67 ±0.97 | 9.83 ±0.52 | 12.40 ±2.23 | 11.76 ±2.26 |
| TRINITY_DN3288_c0_g1_i1 | m.28615 | EDEM3 9606.ENSP00000318147 | 18.19 ±2.00 | 20.65 ±4.00 | 18.54 ±4.16 | 26.65 ±3.49 | 25.72 ±5.54 |

**Table S15.** Histone acetylation proteins in *Leucetta chagosensis*. HAT, histone acetyltransferase; GNAT, GCN5-related N-acetyltransferase; MYST, Moz, Tbf2/Sas3, Sas2, Tip60; HDAC, histone deacetylase.

| | Transcript | Peptide | Domains architecture | UniProt top blastP hit (e-value ≤1 × $10^{-5}$) |
|---|---|---|---|---|
| **HATs** | | | | |
| GNAT | TRINITY_DN697_c1_g1_i1 | m.42160 | zf-TAZ; KIX; Bromodomain; DUF902; HAT_KAT11; ZZ; zf-TAZ | CBP_HUMAN |
| | TRINITY_DN23176_c2_g1_i1 | m.17493 | ELP6 | ELP6_MOUSE |
| | TRINITY_DN6338_c0_g1_i1 | m.15228 | Hat1_N | HAT1_RAT |
| MYST | TRINITY_DN3563_c0_g1_i1 | m.36178 | NuA4 | EAF6_HUMAN |
| | TRINITY_DN15420_c0_g3_i1 | m.4802 | Tudor-knot; MOZ_SAS | KAT5_PONAB |
| | TRINITY_DN1015_c1_g4_i1 | m.16765 | PHD; MOZ_SAS | KAT6A_RAT |
| | TRINITY_DN3840_c0_g1_i1 | m.52795 | Linker_histone; PHD; MOZ_SAS | KAT6B_MACFA |
| | TRINITY_DN5561_c0_g1_i1 | m.47773 | Tudor-knot; MOZ_SAS | KAT8_HUMAN |
| **HDACs** | | | | |
| HDAC | TRINITY_DN6238_c2_g1_i3 | m.19846 | Hist_deacetyl; zf-UBP | HDAC6_MOUSE |
| | TRINITY_DN8343_c0_g1_i2 | m.19433 | Hist_deacetyl | HDAC1_STRPU |
| | TRINITY_DN7404_c0_g1_i1 | m.61391 | Hist_deacetyl | HDAC4_CHICK |
| | TRINITY_DN7210_c0_g1_i2 | m.66788 | Hist_deacetyl | HDAC8_DANRE |
| | TRINITY_DN18742_c0_g1_i1 | m.47973 | Hist_deacetyl | HDAC3_CHICK |
| | TRINITY_DN21933_c0_g1_i6 | m.13499 | Hist_deacetyl | HDA11_HUMAN |
| SIRTUIN | TRINITY_DN11909_c1_g1_i1 | m.21770 | SIR2 | SIR1_HUMAN |
| | TRINITY_DN31771_c0_g2_i3 | m.37999 | SIR2 | HST2_SCHPO |
| | TRINITY_DN918_c0_g1_i1 | m.47300 | SIR2 | SIR2_DANRE |
| | TRINITY_DN12489_c0_g1_i4 | m.12725 | SIR2 | SIR3_HUMAN |
| | TRINITY_DN6868_c0_g1_i1 | m.23105 | SIR2 | SIR6_HUMAN |
| | TRINITY_DN102561_c0_g1_i1 | m.70670 | SIR2 | SIR1_ARATH |
| | TRINITY_DN12382_c0_g1_i8 | m.632 | SIR2 | SIR1_ORYSI |
| | TRINITY_DN12382_c0_g2_i1 | m.634 | SIR2 | SIR1_ARATH |
| | TRINITY_DN7269_c5_g1_i1 | m.66723 | SIR2 | SIR5_DANRE |
| | TRINITY_DN13778_c0_g1_i1 | m.3618 | SIR2 | SIR4_MOUSE |

**Table S16.** Histone methylation proteins in *Leucetta chagosensis*. HMT, histone methyltransferase; HDM, histone demethylase; JMJC, Jumonji-C; LSD, lysine specific demethylase

| | Transcript | Peptide | Domains architecture | UniProt top blastP hit (e-value ≤1 × $10^{-5}$) |
|---|---|---|---|---|
| **HMTs** | TRINITY_DN22125_c0_g1_i1 | m.24761 | zf-CXXC; PHD; zf-HC5HC2H; FYRN; FYRC; SET | KMT2A_MOUSE |
| | TRINITY_DN5577_c0_g1_i1 | m.47894 | zf-HC5HC2H_2; FYRN; FYRC; SET | KMT2D_MOUSE |
| | TRINITY_DN2306_c0_g1_i1 | m.17137 | PHD; SET | KMT2E_MOUSE |
| | TRINITY_DN52819_c0_g1_i4 | m.4269 | SET; Bromodomain; BAH | ASH1L_MOUSE |
| | TRINITY_DN1773_c1_g1_i4 | m.40396 | SET | ATXR4_ARATH |
| | TRINITY_DN7965_c0_g1_i1 | m.7502 | SET; SET | SETB1_DROME |
| | TRINITY_DN1828_c1_g1_i3 | m.11523 | Ank_2; Ank_2; Pre-SET; SET | EHMT1_HUMAN |
| | TRINITY_DN8244_c0_g2_i1 | m.66409 | Ank_2; Ank_4; Ank_2; Pre-SET; SET | EHMT1_MOUSE |
| | TRINITY_DN8131_c0_g2_i2 | m.50394 | SET | EZH2_HUMAN |
| | TRINITY_DN37009_c0_g1_i2 | m.56869 | SET | NSD3_MOUSE |
| | TRINITY_DN2800_c0_g1_i1 | m.54005 | SET | SE1BA_DANRE |
| | TRINITY_DN3301_c1_g1_i3 | m.23728 | SET; WW; SRI | SETD2_MOUSE |
| | TRINITY_DN45292_c0_g1_i1 | m.35956 | SET; Rubis-subs-bind | SETD3_PAPAN |
| | TRINITY_DN2603_c0_g2_i1 | m.8640 | SET | SETMR_RAT |
| | TRINITY_DN3730_c0_g1_i3 | m.17779 | SET | S421A_XENLA |
| | TRINITY_DN6256_c0_g1_i15 | m.19877 | SET | SET8A_DANRE |
| | TRINITY_DN13440_c0_g1_i1 | m.52207 | SET | SET1_CAEEL |
| | TRINITY_DN17205_c0_g1_i2 | m.2189 | SET | SMYD4_PONAB |
| | TRINITY_DN19051_c0_g4_i1 | m.56216 | SET | SMYD4_MOUSE |
| | TRINITY_DN16429_c0_g2_i1 | m.38199 | SET | SMYD5_XENLA |
| **HDMs** | | | | |
| JMJC | TRINITY_DN218_c2_g1_i7 | m.67119 | JmjC | JMD6B_XENLA |
| | TRINITY_DN15944_c0_g1_i4 | m.18504 | PHD; JmjC | PHF8_HUMAN |
| | TRINITY_DN17239_c0_g1_i1 | m.2239 | PHD; JmjC | PHF8_DANRE |
| | TRINITY_DN90420_c0_g1_i1 | m.20127 | JmjC; zf-CXXC; F-box-like | KDM2A_XENTR |
| | TRINITY_DN2182_c0_g1_i1 | m.1188 | JmjC | KDM3B_MOUSE |
| | TRINITY_DN46962_c0_g1_i5 | m.15661 | JmjC | KDM3B_HUMAN |
| | TRINITY_DN25095_c0_g1_i18 | m.9243 | JmjN; JmjC; PHD_2; zf-HC5HC2H_2 | KDM4A_MOUSE |
| | TRINITY_DN8235_c0_g1_i2 | m.66253 | JmjN; ARID; PHD; JmjC; zf-C5HC2; PLU-1; PHD; PHD | KDM5A_HUMAN |
| | TRINITY_DN1517_c0_g1_i7 | m.53254 | JmjN; ARID; PHD; JmjC; zf-C5HC2; PLU-1 | KDM5B_MOUSE |
| | TRINITY_DN2610_c0_g4_i1 | m.8652 | JmjC | KDM6A_MOUSE |
| LSD | TRINITY_DN7999_c2_g1_i1 | m.7496 | SWIRM; Amino_oxidase; Amino_oxidase | KDM1B_HUMAN |

**Table S17.** DNA methylation machinery components in *Leucetta chagosensis*. DNMT, DNA methyltransferase; UHRF1, ubiquitin-like with PHD and RING finger domains 1; TET, ten-eleven translocation enzyme; TDG, thymine DNA glycolase.

| | Transcript | Peptide | Domains architecture | UniProt top blastP hit (e-value ≤1 × $10^{-5}$) |
|---|---|---|---|---|
| DNMT1 | TRINITY_DN14345_c0_g1_i1 | m.59466 | DMAP_binding; DNMT1-RFD; zf-CXXC; BAH; BAH; DNA_methylase | DNMT1_PARLI |
| UHRF1 | TRINITY_DN28136_c0_g3_i1 | m.21042 | ubiquitin; TTD; PHD; SAD_SRA | UHRF1_XENLA |
| TET | TRINITY_DN2812_c3_g1_i3 | m.53847 | zf-CXXC; Tet_JBP | TET2_HUMAN |
| TDG | TRINITY_DN13318_c0_g1_i1 | m.60464 | UDG | UNG_HUMAN |
| | TRINITY_DN4868_c1_g2_i3 | m.57642 | UDG | TDG_HUMAN |

**Table 18.** Epigenetic modifiers in module L5 hub genes. Gene significance was based on gene's correlation value with RCP 8.5. GS, gene significance; MM, module membership; HAT, histone acetyltransferase; GNAT, GCN5-related N-acetyltransferase; HDAC, histone deacetylase; SIRT, sirtuin; HMT, histone methyltransferase; HDM, histone demethylase; JMJC, Jumonji-C.

| | Transcript | Peptide | GS | MM | UniProt top blastP hit (e-value ≤1 × $10^{-5}$) |
|---|---|---|---|---|---|
| HAT (GNAT) | TRINITY_DN697_c1_g1_i1 | m.42160 | 0.69 | 0.94 | CBP_HUMAN |
| | TRINITY_DN23176_c2_g1_i1 | m.17493 | 0.51 | 0.91 | ELP6_MOUSE |
| HDAC (SIRT) | TRINITY_DN918_c0_g1_i1 | m.47300 | 0.62 | 0.91 | SIR2_DANRE |
| | TRINITY_DN6868_c0_g1_i1 | m.23105 | 0.72 | 0.82 | SIR6_HUMAN |
| HMT | TRINITY_DN2306_c0_g1_i1 | m.17137 | 0.45 | 0.85 | KMT2E_MOUSE |
| | TRINITY_DN7965_c0_g1_i1 | m.7502 | -0.53 | -0.94 | SETB1_DROME |
| | TRINITY_DN8244_c0_g2_i1 | m.66409 | -0.45 | -0.91 | EHMT1_MOUSE |
| HDM (JMJC) | TRINITY_DN46962_c0_g1_i5 | m.15661 | -0.58 | -0.94 | KDM3B_HUMAN |
| | TRINITY_DN8235_c0_g1_i2 | m.66253 | 0.66 | 0.82 | KDM5A_HUMAN |

**Table S19.** Transcription factors in module L5 hub genes. Transcription factors were annotated through blastP (e-value < $1x10^{-5}$) against the human proteome v 11.5 from STRING v.11 database (44). Gene significance was based on correlation value with RCP 8.5. Only genes that were differentially expressed (FDR < 0.05) at RCP 8.5 were included. GS/MM, Gene significance/Module membership; FC, Fold change; FDR, False Discovery Rate.

| Transcript | Peptide | GS/MM | $log_2$FC (FDR) | Pfam | Annotation |
|---|---|---|---|---|---|
| TRINITY_DN4418_c1_g2_i11 | m.46049 | 0.58/0.93 | 0.91 (4.75E-02) | bZIP | *CREB3L1* 9606.ENSP00000481956 |
| TRINITY_DN105129_c0_g1_i1 | m.68190 | 0.62/0.93 | 0.90 (2.58E-02) | bZIP | *CEBPB* 9606.ENSP00000305422 |
| TRINITY_DN96882_c0_g1_i1 | m. 6568 | 0.65/0.94 | 1.09 (5.04E-03) | bZIP | No match |
| TRINITY_DN2650_c0_g1_i1 | m.8707 | 0.78/0.94 | 1.32 (7.02E-04) | bZIP | *XBP1* 9606.ENSP00000216037 |
| TRINITY_DN60042_c0_g1_i2 | m.10477 | 0.38/0.92 | 1.11 (9.30E-03) | bZIP | *XBP1* 9606.ENSP00000216037 |
| TRINITY_DN97884_c0_g1_i1 | m.31859 | 0.65/0.81 | 1.41 (5.39E-03) | bZIP_Maf | *NRL* 9606.ENSP00000454062 |
| TRINITY_DN300_c5_g1_i1 | m.5800 | -0.62/-0.90 | -1.65 (4.55E-02) | HLH | *HEY2* 9606.ENSP00000357348 |
| TRINITY_DN29299_c1_g1_i1 | m.16624 | -0.76/-0.85 | -2.90 (2.91E-07) | HLH | *HAND2* 9606.ENSP00000352565 |
| TRINITY_DN3682_c0_g1_i2 | m.43816 | -0.68/-0.84 | -1.10 (9.01E-03) | HLH | *TFAP4* 9606.ENSP00000204517 |
| TRINITY_DN85094_c0_g1_i1 | m.11871 | 0.53/0.93 | 1.41 (2.93E-04) | HMG_box | *SOX9* *9606.ENSP00000245479* |
| TRINITY_DN16966_c0_g1_i1 | m.32901 | 0.49/0.86 | 5.31 (1.92E-12) | HMG_box | *SOX10* 9606.ENSP00000380093 |
| TRINITY_DN16966_c0_g3_i1 | m.32904 | 0.50/0.90 | 7.80 (4.70E-16) | HMG_box | *SOX14* 9606.ENSP00000305343 |
| TRINITY_DN16966_c0_g2_i1 | m.32903 | 0.44/0.92 | 9.07 (3.44E-14) | HMG_box | *SOX6* 9606.ENSP00000379644 |
| TRINITY_DN3467_c0_g1_i3 | m.44348 | 0.55/0.95 | 1.96 (1.11E-05) | Homeobox | *ALX4* 9606.ENSP00000332744 |
| TRINITY_DN6222_c1_g1_i3 | m.19918 | 0.72/0.84 | 6.15 (1.25E-07) | Homeobox KN | No match |
| TRINITY_DN22859_c0_g1_i7 | m.16256 | 0.76/0.88 | 5.38 (1.14E-08) | Homeobox KN | No match |
| TRINITY_DN311_c0_g2_i2 | m.5745 | 0.70/0.95 | 0.84 (3.32E-02) | HTH | *EDF1* 9606.ENSP00000224073 |
| TRINITY_DN13956_c0_g2_i1 | m.46205 | 0.24/0.81 | 3.53 (1.15E-05) | Pou; Homeobox | *POU1F1* 9606.ENSP00000342931 |
| TRINITY_DN123341_c0_g1_i1 | m.70023 | 0.34/0.87 | 1.15 (9.72E-03) | zf-C2H2 | *KFL10* 9606.ENSP00000285407 |
| TRINITY_DN102497_c0_g1_i1 | m.69137 | 0.33/0.81 | 1.43 (8.32E-04) | zf-C2H2 | *EGR1* 9606.ENSP00000239938 |

**Table S20.** Structural homologs of *XBP1* dimer pairs. QMEANDisCO is a composite scoring function derived from the entire structure and per residue quality estimates (0, lowest - 1, highest).

| Dimer | Best hit structural homolog | Sequence identity (%) | QMEANDisCO |
|---|---|---|---|
| *ScerHAC1* homodimer | Transcription factor MafA (4eot.1.A) | 26.53 | 0.76 ± 0.08 |
| *AthaBZIP60* homodimer | Chicken CREB3 bZIP (6iak.1.A) | 31.88 | 0.69 ± 0.07 |
| *HsapXBP1* homodimer | Transcription factor MafB (2wty.1.A) | 26.39 | 0.65± 0.07 |
| *LchaXBP1_1* homodimer | Transcription factor MafB (2wty.1.A) | 26.39 | 0.67 ± 0.07 |
| *LchaXBP1_2* homodimer | Transcription factor MafG (3a5t.1.A) | 36.99 | 0.57 ± 0.07 |
| *LchaXBP1* heterodimer | Transcription factor FosB/JunD (5vpd.2) | 33.33 | 0.66 ± 0.08 |
| *PoriXBP1_1* homodimer | Transcription factor MafA (4eot.1.A) | 26.39 | 0.64 ± 0.07 |
| *PoriXBP1_2* homodimer | Transcription factor MafB 2wty.1.A) | 26.03 | 0.63 ± 0.07 |
| *PoriXBP1* heterodimer | Transcription factor FosB/JunD (7ucc.1) | 29.91 | 0.67 ± 0.08 |
| *CspXBP1_1* homodimer | Transcription factor MafB (2wty.1.A) | 29.58 | 0.69 ± 0.07 |
| *CspXBP1_2* homodimer | Transcription factor MafA (4eot.1.A) | 27.40 | 0.67 ± 0.07 |
| *CspXBP1* heterodimer | Transcription factor FosB/JunD (7ucc.1) | 32.79 | 0.64 ± 0.07 |

**Table S21.** Top docking conformation for *XBP1*-CRE complexes. VDW, van der Waals energy.

| Complex | Conformation code | Electrostatics | Desolvation | VDW | PyDockDNA |
|---|---|---|---|---|---|
| *ScerHAC1* homodimer-CRE | 5086 | -223.725 | 41.322 | 11.477 | -181.255 |
| *AthaBZIP60* homodimer-CRE | 7576 | -275.059 | 55.220 | 101.253 | -209.714 |
| *HsapXBP1* homodimer-CRE | 8406 | -247.531 | 58.756 | -15.183 | -190.293 |
| *LchaXBP1_1* homodimer-CRE | 728 | -232.382 | 53.842 | -24.946 | -181.035 |
| *LchaXBP1_2* homodimer-CRE | 4140 | -214.038 | 29.529 | -43.182 | -188.827 |
| *LchaXBP1* heterodimer-CRE | 940 | -185.814 | 30.264 | 16.221 | -153.928 |
| *PorXBP1_1* homodimer-CRE | 26 | -232.788 | 58.739 | -15.701 | -175.619 |
| *PoriXBP1_2* homodimer-CRE | 881 | -251.396 | 38.571 | -28.962 | -215.721 |
| *PoriXBP1* heterodimer-CRE | 7404 | -163.946 | 31.027 | -27.088 | -135.628 |
| *CspXBP1_1* homodimer-CRE | 291 | -231.297 | 59.375 | -17.690 | -173.691 |
| *CspXBP1_2* homodimer-CRE | 395 | -216.015 | 48.198 | -21.629 | -169.980 |
| *CspXBP1* heterodimer-CRE | 3065 | -146.434 | 19.262 | -13.863 | -128.558 |

**Table S22.** Binding potential of *XBP1* dimers to CRE DNA sequence. Polar interactions between specific residues in *XBP1* dimers and cognate DNA sequence are color-coded. DNA bases in bold font are part of the 'aureobox'. Interactions in bold font are polar interactions between an *XBP1* dimer and the aureobox "TGACGT". bZIP-DNA interfaces were identified through PDBePISA (46).

| Dimer | Chain | Basic region residues | Polar interaction details | Chain | DNA bases |
|---|---|---|---|---|---|
| *ScerHAC1* homodimer | C | RRIERILR(N/N)RRAAHQS(R/R)EKKRLH | **ARG'57.C/NH2 – DT'2.A/O4**<br>ASN'49.C/O – DC'3.A/N4<br>ASN'49.C/O – DA'4.A/N6<br>ARG'45.C/NH1 – DG'-7.B/O6 | A<br>B | CCTTGGC**TGACGT**CAGCCAAG<br>CCTTGGC**TGACGT**CAGCCAAG |
| | D | RRIERILRNRRAAHQSREKKRLH | SER'56.C/OG – DC'-5.B/O5'<br>**ARG'57.C/NH2 – DG'-3.B/O6**<br>**ARG'45.D/NH2 – DT'-4.A/O5'** | A<br>B | CCTTGGC**TGACGT**CAGCCAAG<br>CCTTGGC**TGACGT**CAGCCAAG |
| *AthaBZIP60* homodimer | C | KKRRRRV(R/R)(N/N/N/N)RDAAVRSRERKKEY | **ARG'149.C/NH1 – DT'-4.A/O4'**<br>ARG'149.C/NH1 – DC'-5.A/O3';O2<br>**ASN'150.C/ND2 – DG'-3.A/O6**<br>**ARG'160.C/NH1 – DG'-3.A/O3'**<br>**ASN'150.C/O – DC'-1.A/NH4** | A<br>B | CCTTGGC**T(G/G)ACGT**CAGCCAAG<br>CCTTGGC**TGACGT**CAGCCAAG |
| | D | KKRRRRVRNRDAAVRSRERKKEY | **ARG'147.C/NH1 – DA'-2.B/O3'**<br>**ASN'150.C/ND2 – DT'2.B/O4**<br>**ARG'158.C/NH1 – DT'-4.B/O5'**<br>**ASN'150.C/O – DC'-1.B/N4**<br>ASN'150.D/O – DC'6.A/N4 | A<br>B | CCTTGGC**TGACGT**CAGCCAAG<br>CCTTGGC**TGACGT**CAGCCAAG |
| *HsapXBP1* homodimer | C | KALRRKLKNRVAAQTARDRKKAR | LYS'79.C/NZ – DG'-7.B/O3'<br>ARG'76.C/NH2 – DG'-6.B/O6<br>ARG'90.C/NH2 – DC'-5.B/O3' | A<br>B | CCTTGGC**TGACGT**CAGCCAAG<br>CCTTGG(C/C)**TGACGT**CAGCCAAG |
| | D | KALRRKLKNRVAAQTARDRKKAR | ASN'80.C/OD1 – DC'-5.B/N4<br>ARG'90.D/NH2 – DC'-5.A/O3' | A<br>B | CCTTGGC**TGACGT**CAGCCAAG<br>CCTTGGC**TGACGT**CAGCCAAG |
| *LchaXBP1_1* homodimer | C | KRER(R/R)KLNNRVSAQHARDRKKSY | ARG'42.C/NH2 – DG'-6.A/O6<br>ARG'56.C/NH2 – DG'-6.A/O3<br>ARG'42.C/NH2 – DG'5.B/O6<br>**LYS'58.C/NZ – DA'-2.B/O3'** | A<br>B | CCTTG(G/G)C**TGACGT**CAGCCAAG<br>CCTTGGC**TGACGT**CAGCCAAG |
| | D | KRERRKLNNRVSAQHARDRKKSY | **ARG'54.D/NH1 – DA'-2.A/O5'**<br>**SER'49.D/OG – DT'-4.B/O4**<br>**ARG'56.D/NH2 – DT'-4.B/O3'** | A<br>B | CCTTGGC**TGACGT**CAGCCAAG<br>CCTTGGC**(T/T)GACGT**CAGCCAAG |
| *LchaXBP1_2* homodimer | C | KLKRRKVFNRVSAQIARDRRKNY | **ARG'38.C/NE – DC'1.A/O3'**<br>**ARG'45.C/NE – DA'-2.A/O5'**<br>ARG'47.C/NH2 – DC'-5.B/O3' | A<br>B | CCTTGGC**TGACGT**CAGCCAAG<br>CCTTGGC**TGACGT**CAGCCAAG |
| | D | KLKRRKVFNRVSAQIARDRRKNY | **ARG'33.D/NH2 – DG'5.B/O6**<br>**ARG'38.D/NH2 – DG'1.B/O3'**<br>ARG'45.D/NE – DA'-2.B/O3' | A<br>B | CCTTGGC**TGACGT**CAGCCAAG<br>CCTTGGC**TGACGT**CAGCCAAG |
| *PoriXBP1_1* homodimer | C | KRER(R/R)KLN(N/N)RVSAQHARDRKKSY | ALA'56.D/N - DG'-6.A/O5'<br>**ARG'57.D/NH2 - DT'-4.A/O4**<br>**ARG'57.D/NH2 - DG'-3.A/O6**<br>SER'52.D/OG - DC'-5.A/N4 | A<br>B | CCTTGGC**TGACG(T/T)**CAGCCAAG<br>CCTTGGC**TGACGT**CAGCCAAG |
| | D | KRERRKLNNRVSAQHA(R/R)DRKKSY | **ARG'45.C/NH2 - DT'2.A/O4**<br>**ASN'49.C/ND2 - DT'2.A/O4**<br>**ARG'45.C/NH2 - DG'-3.B/O6**<br>**ASN'49.C/OD1 - DA'-2.B/N6** | A<br>B | CCTTGGC**TGACGT**CAGCCAAG<br>CCTTGGC**TGACGT**CAGCCAAG |
| *PoriXBP1_2* homodimer | C | KLKRRKMFNRVSAQTARDRRKNY | **ARG'45.D/NH1 - DA'-2.A/O5'**<br>ARG'33.C/NH2 - DG'5.B/O6<br>**LYS'49.C/NZ - DA'-2.B/O3'** | A<br>B | CCTTGGC**TGACGT**CAGCCAAG<br>CCTTGGC**TGACGT**CAGCCAAG |
| | D | KLKRRKMFNRVSAQTARDRRKNY | **SER'40.D/OG - DT'-4.B/O4**<br>ARG'47.D/NH1 - DC'-5.B/O3'<br>ARG'47.C/NH2 - DG'-6.A/O3' | A<br>B | CCTTGGC**TGACGT**CAGCCAAG<br>CCTTGGC**TGACGT**CAGCCAAG |
| *CspXBP1_1* homodimer | C | KRER(R/R)KLNNRVSAQNARDRKKNY | **ARG'54.D/NH1 - DA'-2.A/O5'**<br>ARG'42.C/NH2 - DG'5.B/O6<br>**LYS'58.C/NZ - DA'-2.B/O3'** | A<br>B | CCTTG(G/G)C**TGACGT**CAGCCAAG<br>CCTTGGC**TGACGT**CAGCCAAG |
| | D | KRERRKLNNRVSAQNARDRKKNY | **SER'49.D/OG - DT'-4.B/O4**<br>ARG'42.C/NH2 - DG'-6.A/O6<br>ARG'56.C/NH2 - DG'-6.A/O3' | A<br>B | CCTTGGC**TGACGT**CAGCCAAG<br>CCTTGGC**TGACGT**CAGCCAAG |
| *CspXBP1_2* Homodimer | C | KLHRRKVINRVCAQTSRDRRKNY | **ARG'48.C/NH1 - DG'-3.B/O6**<br>**ARG'36.D/NH2 - DG'-7.B/O6**<br>ARG'48.D/NH1 - DG'-3.B/O6 | A<br>B | CCTTGGC**TGACGT**CAGCCAAG<br>CCTTGGC**TGACGT**CAGCCAAG |
| | D | KLHRRKVINRVCAQTS(R/R)DRRKNY | ARG'50.D/NH2 - DG'-7.B/O3'<br>**ARG'36.C/NH1 - DT'-4.A/O4**<br>**ARG'48.D/NH1 - DT'2.A/O4** | A<br>B | CCTTGGC**TGACGT**CAGCCAAG<br>CCTTGGC**TGACGT**CAGCCAAG |

**Data S1.** Aligned and trimmed amino acid sequences corresponding to the bZIP domain (PF00170) that were included for phylogenetic comparisons of *XBP1* homologs.

>GcomXBP1_3
SEKERTARRKITNRVSAQAARERKKNYITNLEEMIRNLK-------
KDNERIKASSMQLQENLIGLREENGRL

>GcomXBP1_4
SEKERTARRKITNRVSAQAARERKKNYITNLEEMIRNLK-------
KDNERIKASSMQLQENLIGLREENGRL

>ScilXBP1_2
SEKERIQRRKITNRVSAQAARERKKVYVDQLESLVRQLQ-------
NENGKLKSRVGLLETRINIVR------

>LcomXBP1_3
TEPEKRQRRKQNNRVSAQHARDRKKKYIEKLEEQIRNLQ-------
AENFSLRTSEQVLKMQLLGPESDSTI-

>ScilXBP1_1
TEPEKRQRRKQNNRVSAQHARDRKKKYIEKLEEQIRNLQ-------
AENFSLRTSEQVLKMQLLGPE------

>GcomXBP1_1
TPLQKRARRKYNNRVSAQHARDRKKRYVEQLEQEVELLK-------
QENSCLRSSNHKLVAEMQQTKDHNNQL

>GcomXBP1_2
TPLQKRARRKYNNRVSAQHARDRKKRYVEQLEQEVELLK-------
QENSCLRSSNHKLVAEMQQTKDHNNQL

>LcomXBP1_1
SDTEKRQRRKRNNRVSAQHARDRKKQHQEDTERELRALR-------
AENASLKQRNSDLTNLIKECKEERQDL

>CspXBP1_1
TTQEKRERRKLNNRVSAQNARDRKKNYLEDLERIVASLR-------
AENSELKSTNASLTASIDEVRKANESL

>LchaXBP1_1
SFQEKRERRKLNNRVSAQHARDRKKSYLEDLERLVASLK-------
AENTALKSTNASLSSSIQEVRRANESL

>PoriXBP1_1
SFQEKRERRKLNNRVSAQHARDRKKSYLEDLERLVASLK-------
AENAALKSTNASLSCSIQEVRQVNESL

>CspXBP1_2
TPEEKLHRRKVINRVCAQTSRDRRKNYVEQLERLVATLR-------
AENETLKSDNRSLQTSLESLRKQNCLL

>LchaXBP1_2
SPEEKLKRRKVFNRVSAQIARDRRKNYLDRLERLVATLR-------
AENEALVVDNRSLNLSLTNLREEHIRL

>PoriXBP1_2
TPEEKLKRRKMFNRVSAQTARDRRKNYVERLERLVATLR-------
AENEAMVVDNRSLSSLLACLREENNVL

>AthaBZIP60
DAVAKKRRRRVRNRDAAVRSRERKKEYVQDLEKKSKYLERECLRLGRMLECFVAENQ
SLRYCLQK--------

>ScerHAC1
-KEQRRIERILRNRRAAHQSREKKRLHLQYLERKCSLLE-------NLL-----------------------

>NcomXBP1_1
TMEERREKRKLMNRVAAQNARDRKRLYLESLEKKVADLE-------
TQNELLMAENSQLKAQSNILETEMKQL

>AqueXBP1
DESDKREKKKMMNRIAAQNARDRKKNYLESLEKKLALLE-------
EENKKLKEENASLKSETVTLSA-----

>HtubXBP1
TEMDRKEKRRMMNRVAAQNARDRKKQHMESIEAKLALLE-------
QQNRALALENQELKQRTSALEAENESL

>PficXBP1
TEMEKKEKRRMMNRVAAQTARDRKKQYVVELEKKLAQLE-------
DQNKCLVKENEQLKQHTSLLESEKLL-

>CintXBP1_3
ITAIHEIRRRGKNRIAAQRCRKRKMDCIRSLQCQLEQLR-------
EEHLNLMGERRTCQDKSLKLAEMFQKR

>CintXBP1_2
QTLIKDIRRRGKNKVAAQNCRKRKIETITTMEEDVDVLR-------
GRKNDLEMEQDELEARKQNLKSQYNAL

>CintXBP1_4
QELIKSERKRLRNRVAASKCRKRKLERISRLEDKVNNLK-------
NQNLELTSSANLLRQQVAELKSKVMTH

>CintXBP1_1
EKSLKKVRRKIKNKISAQESRRKKKEYVETLEKRMDVYN-------
RENTELRHKLDSLESSNRSLLSQLKSL

>DavaXBP1_1
ERAEKQLKRKMRNRVASQNTRDKRRQYVTDLELQVQRLQ-------
EKNEALSRENQLLKQRTDSLLSANTIL

>DavaXBP1_4
ERAEKQLKRKMRNRVASQNTRDKRRQYVTDLELQVQRLQ-------
EKNEALSRENQLLKQRTDSLLSANTIL

>MleyXBP1
-PDERNLKRKLKNRMSAQQARDRKKLYVSELEERVAQLE-------
KEGIPAQEPFTKTVGQCTIST------

>PbacXBP1
-PEERTLKRKLKNRMSAQQARDRKKVFVSELEGRIAQLE-------
KENENLRRICSMQLGGTGDSA------

>TcasXBP1
-WEEKLQRKKLKNRVAAQTSRDRKKAKMDQMEKALQELF-------
SKNEVLVQECERLKGLNERLSA-----

>DmelXBP1
-WEEKVQRKKLKNRVAAQTSRDRKKARMEEMDYEIKELT-------
DRTEILQNKCDSLQAINESLL------

>DavaXBP1_5
DPIERENRRKIRNRVASQTSRDRKKQHMEDLEKRIELLE-------
EMNQHLIEQNELLRKRSNILEEQNASL

>LcomXBP1_5
TAEEKLNRRKLKNRIAAQTARDRKKLKMHSLEETTESMQ-------
QEREALFNENEDLRLENDRLQEEREEL

>SpurXBP1
STEEKMNRRKLKNRVAAQTARDRKKAQFDDLEAVTSVME-------
ARNKQLLMENSLLKKQNKSLLE-----

>NcomXBP1_3
TPQERIIRRKLKNRVAAQTARDRKKERLMNLEKVVGQLE-------
KENKELKKSNDELRLNMDYLMQQNNML

>HmagXBP1_2
-PQERMIRRKLKNRVAAQSARDRKRERMTELEQIVSRLE-------
NENKELKKSNEELKSSMAYLME-----

>BfloXBP1
TPEEKAMRRKLKNRVAAQTARDRKKAKMDELEVIVAKLE-------
AQNKALQQQNSSLKQQSTSLKM-----

>LgigXBP1
SPDEKFLRRKLKNRVAAQTARDRKKALMSEMEIKIAQLE-------
AEKKKLQDENTTLKIQSDS--------

>NcomXBP1_2
TAEERMLRRKLKNRVAAQTARDRKKARMDELEELVQRLE-------
NENELLKRNNEELRSQMNSLTQENSYL

>DavaXBP1_3
--EERILRRKLKNRVAAQSARDRKKAHMYQMEITLLRVE-------KE-------------------------

>HmagXBP1_1
-VDERILRRKLKNRVAAQTARDRKKAHMEDLETCLARIE-------
KENKFLKKSNQELRSQIHNLNE-----

>TadhXBP1
-PEERRFNRKIKNRIAAQAARDKKKELMNYLESNVDVLR-------
NENQKLRNENDMLKIKIEQLSQ-----

>OcarXBP1
-EDEKLSRRKIKNRVAAQNARDRKRQKMDDMETYVEFLR-------
KQNDELLKENALLKRSLREKEA-----

>CeleXBP1
SQEEKMDRRKLKNRVAAQNARDKKKERSAKIEDVMRDLV-------
EENRRLRAENERLRRQNKNLMNQQNE-

>DavaXBP1_2
TPDEKNLRRKLKNRVAAQYARDKKKAMFEELEGRVSELE-------AFNDQIVRENAKLKE--
----------

>CcanXBP1
SDDEKQLRRKLKNREAAQSARDRKKARMDELEKVVKELQ-------
QQNEALTRENHALKARLQQSNTENSQL

>DpulXBP1
-EQEKFLRRKMKNRVAAQTARDKKKAKMDELEDVVINLR-------
AENNRLKAENQQLLAENARLSG-----

>PpacXBP1
TPEEKLARRKMKNRVAAQTARDRKKERTFGLEDCVKDLV-------
DENKRLKEENEKLMERLNRLEEEAAAR

>HrobXBP1
SHEEKIMRRKLKNRVAAQTARDRKKQRMTELEIAIVALQ-------
EQNKQLETENSALKNCTEALLKENKQL

>LcomXBP1_4
SADEKILRRKLKNRVAAQTARDRKKALMTDLEEQVTKLQ-------
EDNKRLAKENAELRASRVSVQQENQRL

>RnorXBP1
SPEEKALRRKLKNRVAAQTARDRKKARMSELEQQVVDLE-------
EENQKLQLENQLLREKTHGLVIENQEL

>MmusXBP1
SPEEKALRRKLKNRVAAQTARDRKKARMSELEQQVVDLE-------
EENHKLQLENQLLREKTHGLVVENQEL

>BtauXBP1
SPEEKALRRKLKNRVAAQTARDRKKARMSELEQQVVDLE-------
EENQKLLLENQLLREKTHGLVVENQEL

>HsapXBP1
SPEEKALRRKLKNRVAAQTARDRKKARMSELEQQVVDLE-------
EENQKLLLENQLLREKTHGLVV-----

>LcomXBP1_2
TPDERMMRRKLKNRVAAQTARDRKKTKMQELEEAVTALE-------
EENKRLENENIKLRVKTGTLCQENRSL

>CtelXBP1
-PEERMLRRKLKNRVAAQTARDRKKCQMSDLELMVAELE-------
RENQRLQQENNTLRQVTGSLTK-----

>AdigXBP1
-AEERALRRKLKNRVAAQTARDRKKARMVELEEMVAQLE-------
KENKALRLDNESLRKHTEAVDI-----

>NvecXBP1
TVEERALRRKLKNRVAAQTARDRKKARMQDLEEAVESLE-------
RENKRLREENKRLNKSTESLAI-----

## Literature cited

1. Bolger AM, Lohse M, Usadel B. Trimmomatic: a flexible trimmer for Illumina sequence data. Bioinformatics. 2014;30(15):2114-20.

2. Haas BJ, Papanicolaou A, Yassour M, Grabherr M, Blood PD, Bowden J, et al. De novo transcript sequence reconstruction from RNA-seq using the Trinity platform for reference generation and analysis. Nat Protoc. 2013;8(8):1494.

3. Langmead B, Salzberg SL. Fast gapped-read alignment with Bowtie 2. Nat Methods. 2012;9(4):357-9.

4. Li B, Fillmore N, Bai Y, Collins M, Thomson JA, Stewart R, et al. Evaluation of de novo transcriptome assemblies from RNA-Seq data. Genome Biol. 2014;15(12):553.

5. Smith-Unna R, Boursnell C, Patro R, Hibberd JM, Kelly S. TransRate: reference-free quality assessment of de novo transcriptome assemblies. Genome Res. 2016;26(8):1134-44.

6. Simao FA, Waterhouse RM, Ioannidis P, Kriventseva EV, Zdobnov EM. BUSCO: assessing genome assembly and annotation completeness with single-copy orthologs. Bioinformatics. 2015;31(19):3210-2.

7. Conesa A, Gotz S. Blast2GO: A comprehensive suite for functional analysis in plant genomics. Int J Plant Genomics. 2008;2008:619832.

8. Finn RD, Bateman A, Clements J, Coggill P, Eberhardt RY, Eddy SR, et al. Pfam: the protein families database. Nucleic Acids Res. 2014;42(D1):D222-D30.

9. Eddy SR. Profile hidden Markov models. Bioinformatics (Oxford, England). 1998;14(9):755-63.

10. Haas B. Trinotate: transcriptome functional annotation and analysis. 2015. 2015.

11. Srivastava M, Simakov O, Chapman J, Fahey B, Gauthier ME, Mitros T, et al. The Amphimedon queenslandica genome and the evolution of animal complexity. Nature. 2010;466(7307):720-6.

12. Guzman C, Conaco C. Comparative transcriptome analysis reveals insights into the streamlined genomes of haplosclerid demosponges. Sci Rep. 2016;6:18774.

13. Riesgo A, Farrar N, Windsor PJ, Giribet G, Leys SP. The analysis of eight transcriptomes from all poriferan classes reveals surprising genetic complexity in sponges. Mol Biol Evol. 2014;31(5):1102-20.

14. Ryu T, Seridi L, Moitinho-Silva L, Oates M, Liew YJ, Mavromatis C, et al. Hologenome analysis of two marine sponges with different microbiomes. BMC Genomics. 2016;17:158.

15. Pita L, Hoeppner MP, Ribes M, Hentschel U. Differential expression of immune receptors in two marine sponges upon exposure to microbial-associated molecular patterns. Sci Rep. 2018;8(1):16081.

16. Kenny NJ, Francis WR, Rivera-Vicens RE, Juravel K, de Mendoza A, Diez-Vives C, et al. Tracing animal genomic evolution with the chromosomal-level assembly of the freshwater sponge Ephydatia muelleri. Nat Commun. 2020;11(1):3676.

17. Posadas N, Baquiran JIP, Nada MAL, Kelly M, Conaco C. Microbiome diversity and host immune functions influence survivorship of sponge holobionts under future ocean conditions. ISME J. 2021.

18. Fortunato SA, Adamski M, Ramos OM, Leininger S, Liu J, Ferrier DE, et al. Calcisponges have a ParaHox gene and dynamic expression of dispersed NK homeobox genes. Nature. 2014;514(7524):620-3.

19. Voigt O, Fradusco B, Gut C, Kevrekidis C, Vargas S, Wörheide G. Carbonic anhydrases: An ancient tool in calcareous sponge biomineralization. Front Genet. 2021;12.

20. Segata N, Izard J, Waldron L, Gevers D, Miropolsky L, Garrett WS, et al. Metagenomic biomarker discovery and explanation. Genome Biol. 2011;12(6):R60.

21. Emms DM, Kelly S. OrthoFinder: phylogenetic orthology inference for comparative genomics. Genome Biol. 2019;20(1):238.

22. Conway JR, Lex A, Gehlenborg N. UpSetR: an R package for the visualization of intersecting sets and their properties. Bioinformatics. 2017;33(18):2938-40.

23. Bahrami S, Ehsani R, Drablos F. A property-based analysis of human transcription factors. BMC Res Notes. 2015;8:82.

24. Lee KK, Workman JL. Histone acetyltransferase complexes: one size doesn't fit all. Nat Rev Mol Cell Biol. 2007;8(4):284-95.

25. Seto E, Yoshida M. Erasers of histone acetylation: the histone deacetylase enzymes. Cold Spring Harb Perspect Biol. 2014;6(4):a018713.

26. Yi X, Jiang X-J, Li X-Y, Jiang D-S. Histone methyltransferases: novel targets for tumor and developmental defects. Am J Transl Res. 2015;7(11):2159.

27. Kooistra SM, Helin K. Molecular mechanisms and potential functions of histone demethylases. Nat Rev Mol Cell Biol. 2012;13(5):297-311.

28. de Mendoza A, Hatleberg WL, Pang K, Leininger S, Bogdanovic O, Pflueger J, et al. Convergent evolution of a vertebrate-like methylome in a marine sponge. Nat Ecol Evol. 2019;3(10):1464-73.

29. Li B, Dewey CN. RSEM: accurate transcript quantification from RNA-Seq data with or without a reference genome. BMC Bioinformatics. 2011;12:323.

30. Robinson MD, McCarthy DJ, Smyth GK. edgeR: a Bioconductor package for differential expression analysis of digital gene expression data. Bioinformatics. 2010;26(1):139-40.

31. Pfaffl MW. A new mathematical model for relative quantification in real-time RT-PCR. Nucleic Acids Res. 2001;29(9):e45.

32. Thompson KS, Vinson CR, Freire E. Thermodynamic characterization of the structural stability of the coiled-coil region of the bZIP transcription factor GCN4. Biochemistry. 1993;32(21):5491-6.

33. Cohen C, Parry DA. Alpha-helical coiled coils and bundles: how to design an alpha-helical protein. Proteins. 1990;7(1):1-15.

34. Vinson C, Myakishev M, Acharya A, Mir AA, Moll JR, Bonovich M. Classification of human B-ZIP proteins based on dimerization properties. Mol Cell Biol. 2002;22(18):6321-35.

35. Fortunato SAV, Vervoort M, Adamski M, Adamska M. Conservation and divergence of bHLH genes in the calcisponge Sycon ciliatum. Evodevo. 2016;7:23.

36. Fortunato S, Adamski M, Bergum B, Guder C, Jordal S, Leininger S, et al. Genome-wide analysis of the sox family in the calcareous sponge Sycon ciliatum: multiple genes with unique expression patterns. EvoDevo. 2012;3(1):14.

37. Marciniak SJ, Ron D. Endoplasmic reticulum stress signaling in disease. Physiol Rev. 2006;86(4):1133-49.

38. Lin JH, Walter P, Yen TS. Endoplasmic reticulum stress in disease pathogenesis. Annu Rev Pathol. 2008;3:399-425.

39. Wodrich APK, Scott AW, Shukla AK, Harris BT, Giniger E. The Unfolded Protein Responses in Health, Aging, and Neurodegeneration: Recent Advances and Future Considerations. Front Mol Neurosci. 2022;15:831116.

40. Anselmi C, Kowarsky M, Gasparini F, Caicci F, Ishizuka KJ, Palmeri KJ, et al. Two distinct evolutionary conserved neural degeneration pathways characterized in a colonial chordate. Proc Natl Acad Sci U S A. 2022;119(29):e2203032119.

41. Sakarya O, Armstrong KA, Adamska M, Adamski M, Wang IF, Tidor B, et al. A post-synaptic scaffold at the origin of the animal kingdom. PLoS One. 2007;2(6):e506.

42. Hemmrich G, Bosch TC. Compagen, a comparative genomics platform for early branching metazoan animals, reveals early origins of genes regulating stem-cell differentiation. Bioessays. 2008;30(10):1010-8.

43. Jindrich K, Degnan BM. The diversification of the basic leucine zipper family in eukaryotes correlates with the evolution of multicellularity. BMC Evol Biol. 2016;16:28.

44. Mering Cv, Huynen M, Jaeggi D, Schmidt S, Bork P, Snel B. STRING: a database of predicted functional associations between proteins. Nucleic Acids Res. 2003;31(1):258-61.

45. Supek F, Bosnjak M, Skunca N, Smuc T. REVIGO summarizes and visualizes long lists of gene ontology terms. PLoS One. 2011;6(7):e21800.

46. EMBL-EBI. PDBePISA (Proteins, Interfaces, Structures and assemblies) at the European Bioinformatics Institute.
